# miR-301a promotes lung tumorigenesis by suppressing Runx3

**DOI:** 10.1186/s12943-019-1024-0

**Published:** 2019-05-23

**Authors:** Xun Li, Mingtian Zhong, Jiexuan Wang, Lei Wang, Zhanwen Lin, Zhi Cao, Zhujuan Huang, Fengxue Zhang, Yong Li, Ming Liu, Xiaodong Ma

**Affiliations:** 1 Institute for Brain Research and Rehabilitation, Guangdong Key Laboratory of Mental Health and Cognitive Science, Center for Studies of Psychological Application, South China Normal University, Guangzhou, 510631 China; 2 The Research Center of Basic Integrative Medicine, Guangzhou University of Chinese Medicine, Guangzhou, 510006 China; 3 State Key Laboratory of Respiratory Disease, Guangzhou Institute of Respiratory Health, The First Affiliated Hospital of Guangzhou Medical University, Guangzhou Medical University, Guangzhou, 510120 China; 4 Department of Cancer Biology, Lerner Research Institute, Cleveland Clinic, Cleveland, OH USA

**Keywords:** miR-301a, Kras, CD8^+^ T cells, Runx3, IFN-γ

## Abstract

**Background:**

Our previous report demonstrated that genetic ablation of miR-301a reduces *Kras*-driven lung tumorigenesis in mice. However, the impact of miR-301a on host anti-tumor immunity remains unexplored. Here we assessed the underlying molecular mechanisms of miR-301a in the tumor microenvironment.

**Methods:**

The differentially expressed genes were identified by using deep sequencing. The immune cell counts, and cytokines expression were analyzed by realtime PCR, immunohistochemistry and flow cytometry. The role of miR-301a/Runx3 in lung tumor was evaluated on cell growth, migration and invasion. The function of miR-301a/Runx3 in regulating tumor microenvironment and tumor metastasis were evaluated in *Kras* transgenic mice and B16/LLC1 syngeneic xenografts tumor models.

**Results:**

In this work, we identified 1166 up-regulated and 475 down-regulated differentially expressed genes in lung tumor tissues between *Kras*^*LA2*^ and *miR-301a*^*−/−*^*; Kras*^*LA2*^ mice. Immune response and cell cycle were major pathways involved in the protective role of miR-301a deletion in lung tumorigenesis. Overexpression of the miR-301a target, Runx3, was an early event identified in *miR-301a*^*−/−*^*; Kras*^*LA2*^ mice compared to *WT-Kras*^*LA2*^ mice. We found that miR-301a deletion enhanced CD8^+^ T cell accumulation and IFN-γ production in the tumor microenvironment and mediated antitumor immunity. Further studies revealed that miR-301a deficiency in the tumor microenvironment effectively reduced tumor metastasis by elevating Runx3 and recruiting CD8^+^ T cells, whereas miR-301a knockdown in tumor cells themselves restrained cell migration by elevating Runx3 expression.

**Conclusions:**

Our findings further underscore that miR-301a facilitates tumor microenvironment antitumor immunity by Runx3 suppression in lung tumorigenesis.

**Electronic supplementary material:**

The online version of this article (10.1186/s12943-019-1024-0) contains supplementary material, which is available to authorized users.

## Background

Lung adenocarcinoma is the most common NSCLC and develops in the pulmonary mucus-producing cells. Generally, lung adenocarcinoma manifests initially as adenoma hyperplasia, which progresses to bronchio-alveolar carcinoma and finally to invasive adenocarcinoma [1]. Activation of oncogenic *Kras* induces lung adenocarcinoma and its evolution through a series of morphological stages from mild hyperplasia to overt carcinoma. With inactivation of tumor suppressor genes, such as *Trp53* or *Pten*, *Kras* significantly accelerates NSCLC malignancy [2, 3]. In primary cells such as mouse embryonic fibroblast (MEFs), *Kras* activation alone induced cellular senescence; however, it caused cellular transformation when *Trp53* mutation was also present [4]. Either suppression of *Kras* signaling or restoration of *Trp53* function is sufficient to cause regression of lung tumors in mice, supporting the possibility that *Kras* and *Trp53* are therapeutic targets in NSCLC [5]. Interestingly, in mouse models with *Trp53* mutation, restoration of wild-type (WT) *Trp53* inhibits growth of lung adenocarcinoma, but has no effects on adenoma formation [6, 7]. These data suggest that mutation of tumor suppressor genes contributes to the early stages of cell transformation and lung tumorigenesis.

Of more than 1000 microRNAs identified, miR-301a has been reported to be overexpressed in several tumor types, including lung [8–10], colon [11], and pancreatic cancer [12]. Mounting evidence indicates that miR-301a is a potential oncogenic miRNA and contributes to tumor formation [11, 13]. Inhibition of miR-301a reduces anchorage-independent colony formation of lung cancer cells [13]. In the orthotopic model of Lewis lung cancer, overexpression of miR-301a in dendritic cells decreased IFN-γ release from antigen-specific cytotoxic T cells, which shifted the antigen-specific T helper cytokine profile from IFN-γ toward IL-13 and IL-17A [14]. Our previous studies showed that deletion of miR-301a reduces *Kras*-driven lung tumorigenesis in mice, suggesting that miR-301a overexpression promotes lung tumorigenesis [15]. In *Kras*^*LA2*^ mice, miR-301a expression in lung or spleen was highest at 9 weeks of age and started to decline at 13 and 18 weeks. Interestingly, miR-301a expression in spleens was upregulated 9.4-fold, whereas that in lung tumors was upregulated only 2.6-fold. Furthermore, deletion of miR-301a in hematopoietic cells leads to reduced development of colitis-associated colon cancer [15]. In patients with NSCLC, miR-301a is most highly expressed in tumor tissues and is associated with poor differentiation and lymph node metastasis [16]. Collectively, these in vitro and in vivo data indicate that miR-301a has an important role in the tumor microenvironment and tumor metastasis.

Runt-related transcription factor 3 (*RUNX3*) was validated to be a direct target of miR-301a and downregulating of *RUNX3* by miR-301a was demonstrated to promote gastric and colorectal cancer cell proliferation and metastasis is. [11, 17]. As a downstream effector of the transforming growth factor-β (TGF-β), *RUNX3* play a critical role in regulation of tumor cell migration, invasion, and epithelial-to-mesenchymal transition (EMT) [18]. *RUNX3* forms a ternary complex with β-catenin/TCF to inhibit Wnt signaling activity in glioma, gastric and intestinal cancers [19–21]. Overexpression of *RUNX3* was demonstrated to inhibit EMT, which promotes metastasis and loss of *RUNX3* in epithelial cells are sensitized to TGFβ induced EMT [21, 22]. Excessive EMT was observed in lung tissue in Runx3 deficient mice and pharmacologic inhibition of EMT expands life spans of new born mice, which was partially due to downregulation of EMT [23]. In *Kras*-induced lung tumorigenesis, *RUNX3* can activate the p14^ARF^-p53 pathway to inhibit the lung adenoma formation [24].

To determine the exact mechanisms of how miR-301a engages *Kras*-driven lung tumorigenesis, we used RNA deep sequencing to further analyze the molecular mechanisms involved in the role of miR-301a in lung tumorigenesis. We found that the immune response and cell cycle were the major pathways that differed between *Kras*^*LA2*^ and *miR-301a*^*−/−*^*;Kras*^*LA2*^ mice. Notably, we revealed that deletion of miR-301a leads to CD8^+^ T cell accumulation, IFN-γ production, and tumor metastatic suppression by elevating *Runx3*, suggesting that miR-301a is a key regulator of the tumor microenvironment in the initiation of tumorigenesis.

## Methods

### Mice and cell lines

Generation of *miR-301a*^*−/−*^ mice in the C57BL/6X129S hybrid background has been described previously [15]. *Kras*^*LA2*^ mice were purchased from the Jackson Laboratory and bred with *miR-301a*^*−/−*^ mice to obtain *miR-301a*^*−/−*^*;Kras*^*LA2*^ mice. Human noncancerous bronchial cell line 16HBE and adenocarcinoma cell lines A549, NCI-H1975 (H1975) and NCI-H1299 (H1299) were purchased and authenticated from the ATCC. PC-9 cell line was purchased and authenticated from the Cell Bank of Type Culture Collection of Chinese Academy of Sciences. Splenocytes, CD4^+^ T cells, CD8^+^ T cells, CD11b^+^ cells, and CD11c^+^ cells were isolated from spleens or tumors using MACS MicroBeads (Miltenyi Biotec, San Diego, CA, USA) following the manufacturer’s instruction.

### Tumor metastasis models

WT and *miR-301a*^*−/−*^ mice (20–23 g, age 8–10 weeks) were used for B16 or LLC1 tumor metastasis models following an established protocol [25, 26]. 2.5Χ10^5^ B16 or LLC1 cells were injected into WT and *miR-301a*^*−/−*^ mice via the tail vein, and after 24 h, 200 μl lentivirus supernatant with either shRNA-control or shRNA-Runx3 was injected into the tail vein every other day until tissue collection. At 4 weeks post-injection, mice were euthanized by CO_2_ asphyxiation. Lung tissues were removed and fixed with 4% formaldehyde, and lung metastasis sites were evaluated by histological observation.

### RNA preparation and sequencing

Total RNA was extracted using the TRI Reagent and Direct-zol RNA kit (Zymo Research, Irvine, CA, USA) following the manufacturer’s instructions. RNA quality and quantity were assessed on an Agilent Bioanalyzer 2100 to ensure an A260/A280 ratio in the range of 1.8–2.0 and an RNA ratio (28S/18S) > 1.0. DNA was removed with DNase I. Poly(A) RNAs were purified by using the Dynabeads mRNA purification kit (Invitrogen, Carlsbad, CA, USA) according to manufacturer’s instructions. Libraries were sequenced and lung tissue mRNA profiles from *Kras*^*LA2*^ and *miR-301a*^*−/−*^*;Kras*^*LA2*^ mice in duplicate were generated with the High-Seq 2000 Illumina sequencing platform by Cofactor Genomics.

### DEG identification and bioinformatics analysis

The relative transcript abundance was measured in reads per kilobase of exon per million mapped reads (RPKM). DEGs were identified and analyzed by web online service from cofactor genomic company with an unpaired student’s t test with *P* value cutoff of 0.05 and fold change more than 2.0 (upregulation) or less than 0.5 (downregulation). The *P* value obtained for DEG were adjusted by applying false discovery rate (FDR) method to correct for multiple hypothesis testing. The DAVID gene annotation tool was used to analyze the DEG functions according to the gene ontology terms with significance of *P* < 0.05 and false discovery rate < 0.05. IPA was used to identify enriched molecular pathways, gene networks, upstream regulators, canonical pathways, and cellular biological consequences. We considered both direct and indirect relationships that were experimentally observed predicted in mouse species and we used the “stringent” setting to filter molecules and relationships in tissues and cell lines. For canonical pathways, the -log(*P*-value) was derived from the right-tailed Fisher’s exact. Transcription factors and cytokines were extracted from upstream regulator analysis. This analysis identifies causal molecules associated with differential expression using both the significance and the direction of differential expression to specify causal predictions. The Z-scores and *P*-value based on overlap between predicted and observed regulator-regulated genes (Fishers exact test) were calculated [27]. miR-301a target gene prediction analysis was performed using the miRWalk2.0 database.

### Quantitative real-time PCR (qPCR)

qPCR was performed using the SYBR Green Supermix (Bio-Rad, CA, USA) and CFX96 real-time PCR detection system (Bio-Rad). We analyzed each sample in duplicate with β-actin as reference (oligonucleotide sequences are provided in Additional file 1: Table S6). Real-time PCR for miR-301a detection was performed using the TaqMan assay (Thermo Fisher, Waltham, MA, USA).

### Western blotting analysis

For western blotting analyses, soluble proteins were subjected to SDS-polyacrylamide gel electrophoresis before being transferred to polyvinylidene difluoride membranes for blotting. Antibodies for Runx3 (D9K6L), β-catenin (D10A8), integrinα4 (D2E1), Pias3 (#4164), and Pten (D4.3) plus the PathScan multiplex western cocktail (including phosphor-Akt, Phospho-Erk1/2 and Rab11, #5301) were purchased from Cell Signaling, and antibodies for Adam23 (ab28302), Fam107b (ab175148), Slc25α21 (ab167033), and Nkrf (ab168829) were purchased from Abcam. Antibodies for Fibronectin (A12932), Vimentin (A2666), N-Cadherin (A0433), E-Cadherin (A3044), MMP9 (A0289), MMP2 (A6247) and Ki67(A11005) were purchase from ABclonal Technology. β-actin antibody (H11459) was purchased from Sigma. The immunoreactive proteins were visualized with SuperSignal West Dura Chemiluminescent (Thermo Fisher).

### Histology immunohistochemistry and immunofluorescence analyses

Lung tissues were collected at the indicated times and perfused with 10% neutral-buffered formalin. After 24 h fixation, tissues were paraffin-embedded and sectioned (4 μm). For immunofluorescence staining, deparaffinized and rehydrated sections were treated for antigen retrieval using sodium citrate buffer. For immunohistochemical staining, sections were incubated with primary antibody to CD45 (ab10558, abcam), CD3 (ab16669, abcam), CD4 (ab183685, abcam), CD8 (ab4055, abcam) and F4/80 (ab6640, abcam) overnight at 4 °C. Tissue sections were developed using the Ultra Vision Detection System (Thermo Fisher). For the TUNEL assay, deparaffinized sections were incubated with 20 μg/ml proteinase K for 15 minat room temperature, washed with PBS, and incubated with TUNEL reaction mixture (Roche Applied Science) for 1 h at 37 °C in a humidified atmosphere. For immunofluorescence staining to measure Runx3 and β-catenin, lung tissue sections were incubated with anti-Runx3 antibody (D9K6L, 1:200, Cell Signaling) and anti-β-catenin (D10A8, 1:200, Cell Signaling) at 4 °C overnight. After being rinsed with PBS, the sections were incubated with Alexa Fluor-488 goat anti-rabbit (1:100, Invitrogen) or Alexa Fluor-647 donkey anti-mouse (1100, Invitrogen) for 2 h at room temperature. After another PBS rinse, the sections were incubated with 1 μg/ml 4′,6-diamidino-2-phenylindole for 5 min, and images were acquired using an Olympus IX51 fluorescence microscope and cellSens software (version 1.5).

### Elisa

To determine IFN-γ secretion ex vivo, lung tissues were minced, weighed, and incubated with DMEM medium at 37 °C for 48 h. IFN-γ concentration in culture supernatant was measured using the Mouse IFN-γ Platinum ELISA kit (Thermo Fisher) according to the manufacturer’s protocol.

### Flow cytometry

Lung tissues were minced and filtered to make a single-cell suspension. Cells were stained with anti-CD3-FITC (BD PharMingen, San Diego, CA, USA). After fixation and permeabilization, cells were stained with anti-IFN-γ-PE and isotypic mAbs (BD PharMingen). For flow cytometry analysis of B16 metastatic tumor models, single-cell suspensions were stained with anti-CD3-APC, anti-CD4-Alexa Fluor 488, anti-CD8-PE, anti-CD11b-APC, and anti-F4/80-Alexa Fluor488. All events were acquired using CytoFLEX (Beckman Coulter) equipment according to standard procedures.

### RNA interference and miR-301a inhibition

Using Lipofectamine RNAiMax reagent, NSCLC cell lines were transfected with 25 nM siRNA duplexes targeting *PTEN*, *PIAS3*, and *RUNX3*. After 48 h, the cells were collected, and silencing was measured. siRNA duplex oligonucleotides were purchased from Dharmacon (Dharmacon, Lafayette, CO, USA). For miR-301a inhibition in A549 cells, an LNA-anti-miR-301a inhibitor (Exiqon, Qiagen) was used for transfection, and cells were collected after 48 h. The control was the LNA-miRNA Negative Control A; it has no more than 70% homology to any sequence in the human genome.

### Cell proliferation and migration assay

Cell proliferation was determined using the Cell Counting Kit-8 (CCK-8) kit (Dojindo Molecular Technologies, Inc., China) according to the manufacturer’s protocol. For the cell migration assay, A549 cells were suspended in 1% FBS DMEM, incubated at 37 °C for 2 h, and then placed in the upper transwell chambers. The lower chambers were filled with DMEM with 5% FBS. After 24 h, the inserts were removed, and inner side was wiped with a cotton swab. The cells were fixed with 70% ethanol, stained with Giemsa, and counted under light microscopy.

### In vivo administration of *Runx3* shRNA lentivirus

For in vivo knockdown of Runx3, the shRNA1-Runx3 sequence was: AGGAGCGGTCAAACTGGCGG; shRNA2-Runx3: TTGTGAGCGTGAAACTCTTC; shRNA3-Runx3: ATCGAAGGTCGTTGAACCTG; nonsense sequence to Runx3 (shRNA-control): GGTGTGCAGTTGGAATGTA. Sequences were synthesized and ligated into psiF-copGFP vectors (System Biosciences, Mountain View, CA, USA). 293 T cells were seeded in a 10-cm plate and transfected with 5 μg pRSV-rev, 5 μg pMDL-rre, 5 μg pMD2.G, and 10 μg shRNA plasmid construct. Then the cells were cultured at 37 °C for 48 h, and the virus was harvested by centrifuging the cells at 2000 *g* for 10 min followed by filtration through a 0.45 μm filter. Viruses were tittered by serial dilution and transfected into 293 T cells with 8 μg/ml polybrene, and positive cells with green fluorescent protein were counted by flow cytometry after 48 h transfection. Then 1 Χ10^8^ TU/ml lentivirus supernatant was injected into mice via the tail vein, and at the indicated times, lung tissue was collected, and western blotting and fluorescence microscopy were performed to ensure silencing was achieved.

### Statistical analysis

All statistical analysis was carried out using SPSS16.0 software (SPSS Inc. Chicago, IL, USA). An unpaired two-tailed Student’s *t*-test was performed for two-group comparisons and one-way analysis of variance (ANOVA) analysis was performed for multiple group comparisons.

## Results

### Transcriptional signature analysis of lung tissues from *Kras*^*LA2*^ and *miR-301a*^*−/−*^*;Kras*^*LA2*^

**Fig. 1:**
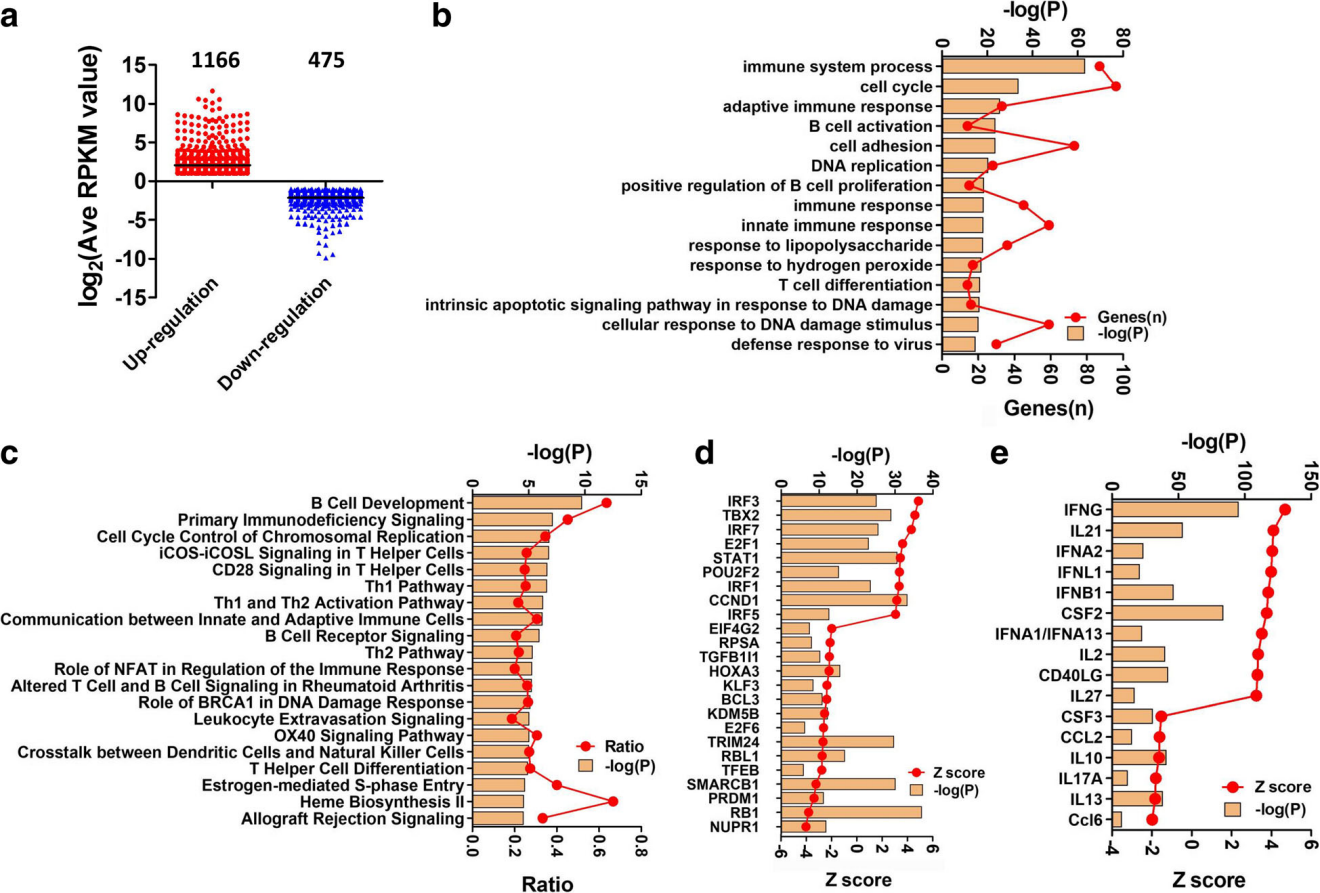
Transcriptional signatures analysis in tumors from *Kras*^*LA2*^ and *miR-301a*^*−/−*^*;Kras*^*LA2*^ mice. (**a**) Number of upregulated and downregulated genes in *miR-301a*^*−/−*^*Kras*^*LA2*^ tumors compared to *Kras*^*LA2*^ tumors. (**b**) Biological functions, (**c**) Canonical pathways, (**d**) Transcription factors and (**e**) Cytokines were categorized

To investigate the role of miR-301a onset lung tumorigenesis, we performed RNA sequencing using total RNA isolated from lung tissues of *Kras*^*LA2*^ and *miR-301a*^*−/−*^*;Kras*^*LA2*^ mice at age of 9 weeks, when the expression of miR-301a is highest [15]. We identified a total of 1641 DEGs (1166 upregulated and 475 downregulated) in lung tissue between *miR-301a*^*−/−*^*;Kras*^*LA2*^ mice and *Kras*^*LA2*^ mice (Fig. 1a, GSE109238). By analyzing the significant biological processes, we found that immune system process and cell cycle were the most significant gene ontology terms, and B cell activation and T cell differentiation were both significantly annotated (Fig. 1b, Additional file 1: Table S1 and Table S2). Ingenuity Pathway Analysis (IPA) revealed the most enriched canonical pathways involved in B cell development, primary immunodeficiency signaling, cell cycle control of chromosomal replication, iCOS-iCOSL signaling in T helper cells, CD28 signaling in T helper cells, and the Th1 pathway (Fig. 1c, Additional file 1: Table S3).

Next, we determined differences in transcription factors and cytokines involved in lung tumorigenesis in *miR-301a*^*−/−*^*;Kras*^*LA2*^ mice compared to *Kras*^*LA2*^ mice. As shown in Fig. 1d, transcription activation factors with high Z-scores were *Irf3*, *Tbx2*, *Irf7*, *E2f1*, *Stat1*, *Pou2f2*, *Irf1*, *Ccnd1* and *Irf5*, whereas the most significant inhibitory transcription factors were *Nupr1*, *Rb1*, *Prdm1*, *Smarcb1*, *Tfeb*, *Rbl1*, *Trim24*, *E2f6*, *Kdm5b*, *Bcl3*, *Klf3*, *Hoxa3*, *Tgfb1l1*, *Prsa* and *Eif4g2* (Fig. 1d). The cytokines with significant activation Z scores are *Ifng*, *Il21*, *Ifna2*, *Ifnl1*, *Ifnb1*, *Csf2*, *Ifna1*, *Il2*, *Cd40lg* and *Il27*, and those with high inhibition Z-scores included *Ccl6*, *Il13*, *Il17a*, *Il10*, *Ccl2* and *Csf3* (Fig. 1e). The most highly implicated IPA network was “DNA Replication, Recombination and Repair, Cell Morphology, and Cancer, Hematological Disease, Immunological Disease.” We merged the total 5 networks related to the cell cycle and immune response pathways and found that IFNG (IFN-γ) and CTNNB1 (β-catenin) were in the core modules within the whole network (Additional file 2: Fig. S1a). Specifically, 283 molecules were identified to be related to lung cancer and the total Z-score of − 0.773 for all 283 molecules was predicted to be inhibited (Additional file 1: Table S4). In addition, 47 molecules were identified to be related to CD8^+^ T lymphocytes, and their predicted activation state was “activated,” with a total Z-score of 2.657 (Additional file 1: Table S5). These results support a role of miR-301a in mediating anti-tumor immune response during lung tumorigenesis.

### miR-301a deficiency recruits T cells and elevates IFN-γ in the tumor microenvironment

**Fig. 2:**
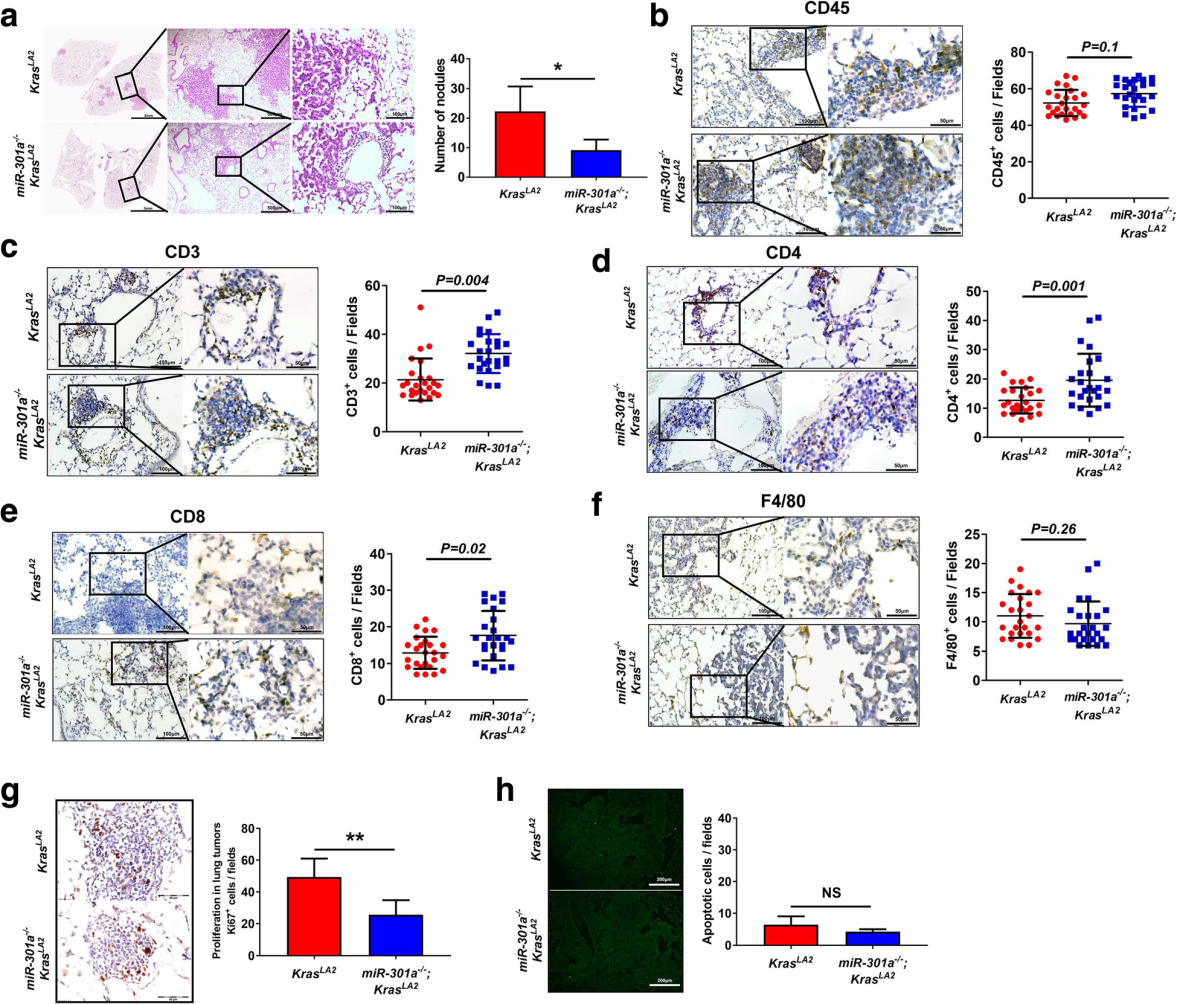
Deletion of miR-301a alters tumor microenvironment. (**a**) Representative hematoxylin and eosin-stained histological sections of lungs isolated from *Kras*^*LA2*^ (*n* = 5) and *miR-301a*^*−/−*^*;Kras*^*LA2*^ (n = 5) mice at 9 weeks of age. Lines highlighted regions of inflammation. Average of tumor nodules were counted on the lung surface of *Kras*^*LA2*^ and *miR-301a*^*−/−*^*;Kras*^*LA2*^ littermates at 9 weeks of age. Quantitative immunohistochemical analysis of leukocytes (**b**: CD45), T cells (**c**: CD3), CD4^+^ T cells (**d**: CD4), CD8^+^ T cells (**e**: CD8), and macrophages (**f**: F4/80) from representative lung adenoma sections; quantitation was restricted to the areas within tumor boundaries. *n* = 25 individual tumors from 5 *Kras*^*LA2*^ or 5 *miR-301a*^*−/−*^*;Kras*^*LA2*^ mice and each individual tumor was staining from 3 serial sections and quantified and averaged as three independent experiments . Values represented the mean ± s.d. of three independent experiments. **P < 0.01 or *P < 0.05 indicates a significant difference between the indicated two groups (two-tailed, unpaired Student’s *t-*test)

To investigate the role of miR-301a in the development of lung cancer, we first compared lung tumor between *Kras*^*LA2*^ and *miR-301a*^*−/−*^*;Kras*^*LA2*^ mice at age of 9 weeks. Consistent with our previous results [15], hematoxylin and eosin (H&E) staining (Fig. 2a) of *miR-301a*^*−/−*^*;Kras*^*LA2*^ lung showed significantly less hyperplasia as well as fewer alveolar adenomas compared with *Kras*^*LA2*^ mutant mice (Fig. 2a). We next evaluated the alteration of infiltrating immune cells in lung tumors. We found no significant difference in the number of CD45^+^ cells in lung tumors between *miR-301a*^*−/−*^*;Kras*^*LA2*^ mice and *Kras*^*LA2*^ mice (Fig. 2b). However, a greater number of CD3^+^, CD4^+^ and CD8^+^ T cells were observed in *miR-301a*^*−/−*^*;Kras*^*LA2*^ mice compared to *Kras*^*LA2*^ mice. (Fig. 2c–e). The number of infiltrating macrophages, as judged by immunostaining with F4/80 antibody, did not significantly differ between the two groups (Fig. 2f). Furthermore, we evaluated cell proliferation and apoptosis in lung tumors. There were significantly fewer Ki67^+^ tumor cells in *miR-301a*^*−/−*^*;Kras*^*LA2*^ mice compared with *Kras*^*LA2*^ mice (Fig. 2g). However, there was no significant difference in cell apoptosis between *miR-301a*^*−/−*^*;Kras*^*LA2*^ mice and *Kras*^*LA2*^ control mice (Fig. 2h).

**Fig. 3:**
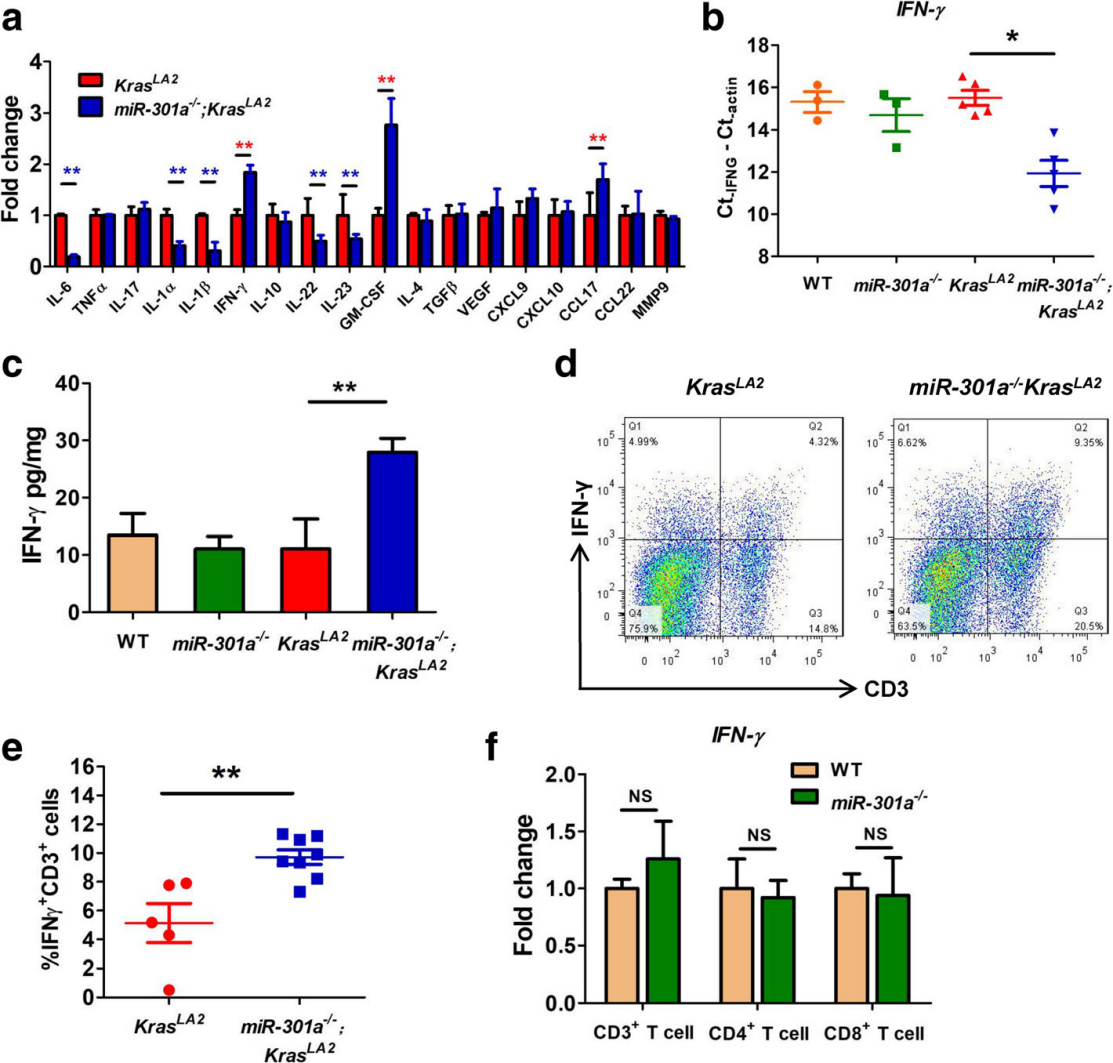
Inhibition of lung tumorigenesis in *miR-301a*^*−/−*^*Kras*^*LA2*^ mice correlates with elevated IFN-γ expression. (**a**) Cytokine gene expression were measured by qPCR in lung tumors isolated from *Kras*^*LA2*^ (n = 5) and *miR-301a*^*−/−*^*Kras*^*LA2*^ (n = 5) mice at 9 weeks of age. (**b**) Relative expression IFN-γ in tumor tissues of WT (*n* = 3), *miR-301a*^*−/−*^(n = 3), *Kras*^*LA2*^ (n = 5), and *miR-301a*^*−/−*^*Kras*^*LA2*^ (n = 5) mice at 9 weeks of age were validated and determined by qPCR. (**c**) Single-cell suspensions were isolated from lung tissues (n = 5 per group), and IFN-γ secretion ex vivo was determined by ELISA. (**d**) Representative results of IFN-γ expression by CD3^+^ T cells isolated from lung tumors in 9-week-old *Kras*^*LA2*^ (n = 5) and *miR-301a*^*−/−*^*Kras*^*LA2*^ mice (*n* = 8). (**e**) The percentage of IFN-γ-positive and CD3-positive cells was counted using flow cytometric analysis of lung tissues from *Kras*^*LA2*^ (n = 5) and *miR-301a*^*−/−*^*Kras*^*LA2*^ (n = 8) mice at 9 weeks of age. (**f**) Relative expression levels of IFN-γ for CD3^+^, CD4^+^ and CD8^+^ T cells of lung tissues from WT (n = 5) and *miR-301a*^*−/−*^ mice (n = 5). Values represented the mean ± s.d. of three independent experiments. **P < 0.01 or *P < 0.05 indicates a significant difference between the indicated groups (two-tailed, unpaired Student’s *t-*test in a, e and f and one-way analysis of variance (ANOVA) in **b** and **c**). NS, not significant

We postulated that miR-301a deletion protects against tumor formation by restraining the pro-inflammatory process. We found significantly lower gene expression of cytokines IL6, IL1α, IL1β, IL22 and IL23 in the lung tumors from *miR-301a*^*−/−*^*;Kras*^*LA2*^ mice than those from *Kras*^*LA2*^ mice (Fig. 3a). Moreover, IFN-γ gene expression validated by qPCR was significantly greater in *miR-301a*^*−/−*^*;Kras*^*LA2*^ lung tissues compared with *Kras*^*LA2*^ lung tissue (Fig. 3a and b), yet IFN-γ level did not differ significantly between WT and *miR-301a*^*−/−*^ lung tissues (Fig. 3b). We noted that from the transcription signature analysis, IFN-γ was the most significantly upregulated cytokine in *miR-301a*^*−/−*^*;Kras*^*LA2*^ lung tissues compared with *Kras*^*LA2*^ lung tissues. As this cytokine is secreted from cells in the lung mucosa-associated lymphoid tissue, we next isolated the whole lung from mice and examined cytokine secretion ex vivo. As shown in Fig. 3c, secretion of IFN-γ increased with miR-301a deletion in Kras-mutated mice. IFN-γ is produced primarily by CD4^+^ T cells, CD8^+^ T cells, and NK cells. To further investigate IFN-γ expression in T cells, we evaluated CD3^+^ T cells by flow cytometric analysis. A higher frequency of IFN-γ producing CD3^+^ T cells was detected in whole-lung tissues from *miR-301a*^*−/−*^*;Kras*^*LA2*^ mice than those from *Kras*^*LA2*^ mice (Fig. 3d and e). In addition, we isolated CD3^+^, CD4^+^, and CD8^+^ T cells from WT and *miR-301a*^*−/−*^ lungs and measured the basal IFN-γ level when miR-301a was deleted. We found that IFN-γ expression did not differ between T cells isolated from lung tissues of WT and *miR-301a*^*−/−*^ mice (Fig. 3f). These results implicate that miR-301a deficiency recruits CD3^+^ T cells to the tumor microenvironment, which leads to higher IFN-γ expression in early lung tumorigenesis.

### miR-301a targets Runx3 in lung tumorigenesis

**Fig. 4:**
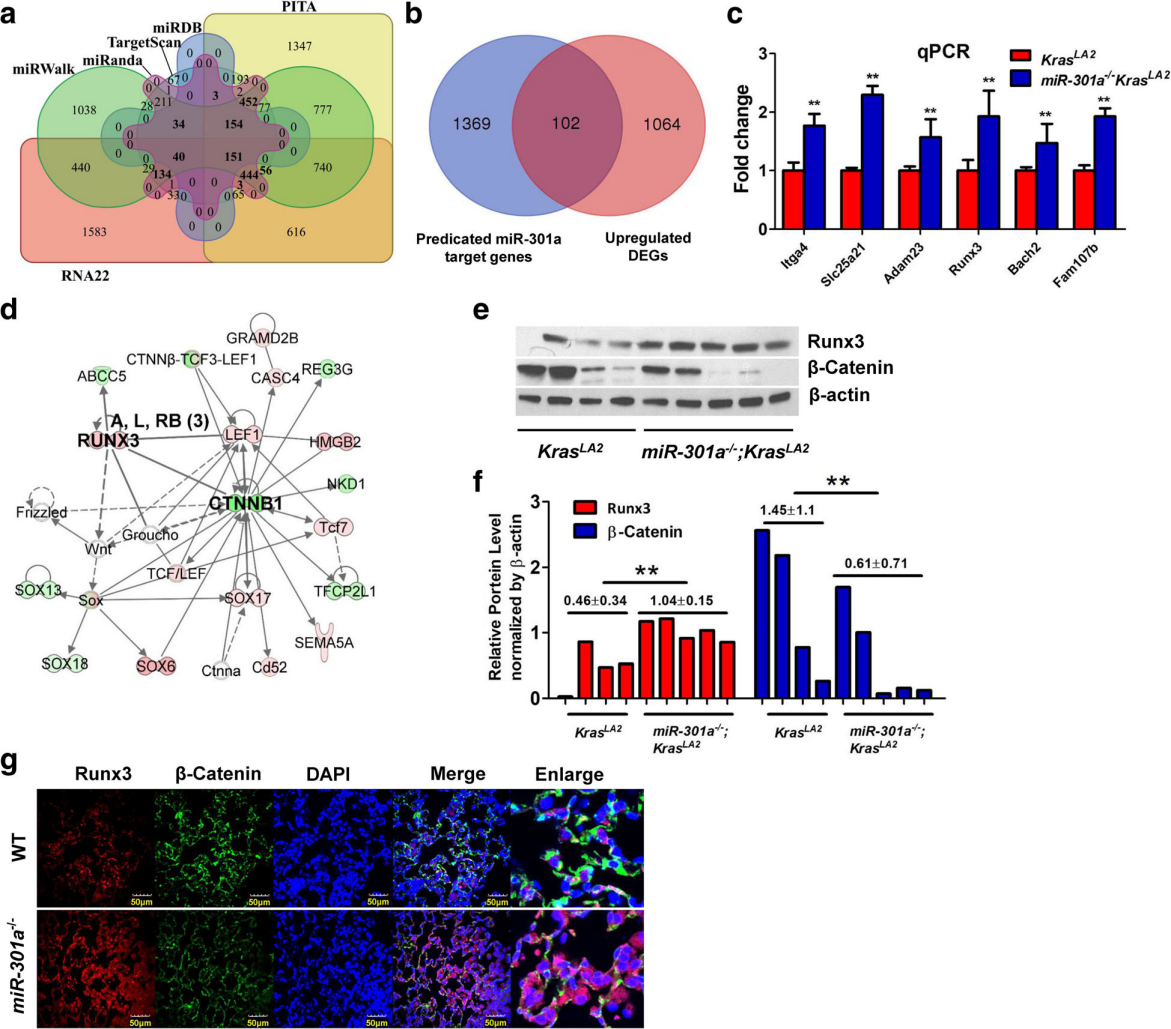
Inhibition of lung tumorigenesis in *miR-301a*^*−/−*^*;Kras*^*LA2*^ mice correlates with elevated Runx3 expression. (**a**) Venn diagram of predicted miR-301a target genes based on analysis of 6 databases (shown in bold). (**b**) Venn diagram of specific genes between predicted miR-301a target genes from (**a**) and DEGs identified by RNA-Seq. (**c**) Real-time PCR validation of 6 significantly upregulated genes from (**b**) (*n* = 3 per group). (**d**) IPA interaction network analysis of significant DEGs identifies the Runx3/β-catenin pathway in lung tumorigenesis with miR-301a deletion. (**e**) Western blotting analyses of Runx3 and β-catenin in the lung. Lung tissues were harvested from *Kras*^*LA2*^ (*n* = 4) and *miR-301a*^*−/−*^*;Kras*^*LA2*^ (n = 5) mice at 9 weeks of age. (**f**) Quantification of the expression of Runx3 and β-catenin from (**e**) using Image J with β-actin as a reference. (**g**) Immunofluorescence analyses of Runx3 and β-catenin in the lung. Lung tissues were from (**e**) and lung sections were stained with antibodies against Runx3 and β-catenin and DAPI (nucleus). Scale bars = 50 μm. Value represented the mean ± s.d. of three independent experiments. ** *P* < 0.01 indicated a significant difference between *Kras*^*LA2*^ and *miR-301a*^*−/−*^*Kras*^*LA2*^ mice (two-tailed, unpaired Student’s *t-*test in c and f)

We used a comprehensive microRNA-target predicting database, miRWalk, which integrate six bioinformatic tools – Targetscan6.2, miRWalk 2.0, miRDB4.0, miRanda-rel2010, RNA22v2, and PITA -- to predict the potential targets of miR-301a. We identified 1471 potential target genes of miR-301a, which were present in more than 4 bioinformatic tools (Fig. 4a). Next, we compared all up-regulated DEGs identified between *Kras*^*LA2*^ and *miR-301a*^*−/−*^*;Kras*^*LA2*^ mice to those 1471 predicted genes. We found that 102 genes overlapped between upregulated DEGs and predicted miR-301a target genes (Fig. 4b). Based on the reads per kilobase of exon per million mapped reads (RPKM) value (*P* < 0.05; log_2_(fold change) > 1) and gene function, we chose 6 tumor suppressor genes (*Itga4*, *Slc25a21*, *Adam23*, *Runx3*, *Bach2* and *Fam107b*) that were significantly upregulated in *miR-301a*^*−/−*^*;Kras*^*LA2*^ lung tissues compared to *Kras*^*LA2*^ lung tissues. As shown by quantitative real-time PCR, upregulation of all 6 genes was significantly greater in lung tissues from *miR-301a*^*−/−*^*;Kras*^*LA2*^ mice than that from *Kras*^*LA2*^ mice (Fig. 4c). These results indicate these 6 genes are potential targets of miR-301a in *Kras-*driven lung tumorigenesis.

IPA interaction network analysis showed that *β-catenin* is present in the core modules in the merged network (Additional file 2: Figure S1a) and indicated that *Runx3* affected the *β-catenin* signaling pathway (Fig. 4d). microRNA-target predicting analysis also revealed a major binding site for miR-301a within the *Runx3* RNA 3’UTR (Additional file 2: Figure S2a). Using a luciferase reporter system, we determined miR-301a directly binds the *Runx3* mRNA and down-regulates Runx3 expression (Additional file 2: Figure S2b and 2c). Next, we determined whether miR-301a deletion affected *Runx3* and *β-catenin* expression in lung tumorigenesis by western blotting and immunofluorescence analyses. As shown in Fig. 4e and f, *Runx3* expression was significantly upregulated, whereas *β-catenin* expression was down-regulated, in lung tissues from *miR-301a*^*−/−*^*;Kras*^*LA2*^ mice than that from *Kras*^*LA2*^ mice. These results were further confirmed by immunofluorescence analysis (Fig. 4g). Taken together, these results indicate that miR-301a directly targets the *Runx3* mRNA, and the protective role of miR-301a deficiency in *Kras*-driven lung tumorigenesis is associated with upregulation of *Runx3* and downregulation of *β-catenin*.

### Inhibition of miR-301a reduces cellular proliferation and migration in NSCLC cell lines

**Fig. 5:**
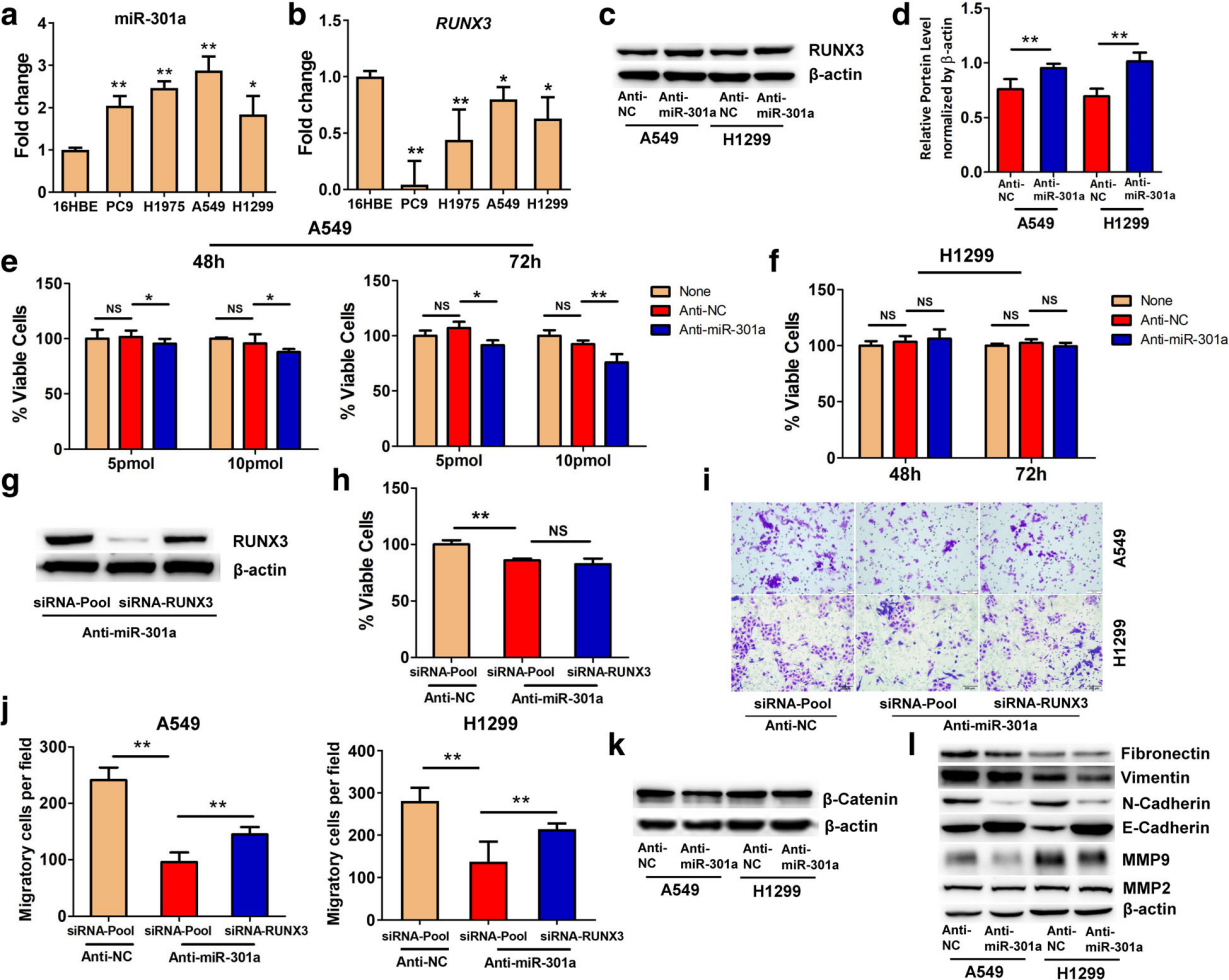
The effects of miR-301a and Runx3 on cell proliferation and migration in A549 and H1299 cells. qPCR analyses of miR-301a (**a**) and its target gene *RUNX*3 (**b**) expression in NSCLC cell lines (PC9, H1975, A549, and H1299) compared to a noncancerous bronchial cell line (16HBE). Total RNAs were extracted from the cell lines and used for reverse transcription and real-time PCR. (**c**) Western blotting analyses of RUNX3 expression in A549 and H1299 cells transfected with anti-negative-control (anti-NC) or LNA-anti-miR-301a (anti-miR-301a). (**d**) Quantification of RUNX3 expression (**c**) using Image J with β-actin as a reference. (**e**) Cell viability of A549 cells transfected with only the transfection reagent, RNAiMAX (None), anti-negative control (anti-NC, 10 pmol), or LNA-anti-miR-301a (anti-miR-301a, 10 pmol). A549 cells were incubated for 48 h and 72 h after transfection, and cell viability was determined by counting cells labeled with CCK-8. (**f**) Cell viability of H1299 cells. H1299 cells were transfected and measured as in (**e**). (**g**) Western blotting shows the efficacy of siRNA knockdown in A549 cells co-transfected with LNA-anti-miR-301a and siRNA-RUNX3 or siRNA-control. (**h**) A549 cells were transfected with anti-NC & siRNA control (siRNA-Pool), anti-miR-301a & siRNA-Pool, or anti-miR-301a & siRNA-RUNX3. At 48 h after transfection, A549 viability was determined as in (**e**). (**i**) Migration of A549 cells was determined with a transwell migration assay. (**j**) The percentage of migratory cells in (**i**). (**k**) Western blotting analyses of β-catenin expression in A549 and H1299 cells transfected with Anti-NC or Anti-miR-301a. (**l**) Western blotting analyses of fibronectin, vimentin, N-cadherin, E-cadherin, MMP9 and MMP2 expression in A549 and H1299 cells transfected with Anti-NC or Anti-miR-301a. Values represented the mean ± s.d. of three independent experiments. **P < 0.01 or *P < 0.05 indicates a significant difference between the indicated groups (two-tailed, unpaired Student’s *t-*test in d and one-way analysis of variance (ANOVA) in others). NS, not significant

To determine whether elevating *RUNX3* by miR-301a deletion alters lung tumor cell phenotype, we first measured the expression of miR-301a and *RUNX*3 in noncancerous lung epithelial cell line 16HBE and NSCLC cell lines PC9, H1975, A549 and H1299. We found that miR-301a was highly expressed in NSCLC cell lines as compared with 16HBE cells as determined by quantitative real-time PCR (Fig. 5a). We noted that of the six genes validated by RNA-sequencing, only *RUNX3* was significantly downregulated in these four NSCLC cell lines as compared with 16HBE cells (Fig. 5b and Additional file 2: Figure S3a and 3b). Next, we chose two NSCLC cell lines, the *Trp53*-wt lung cancer A549 line and *Trp53*-null lung cancer H1299 line, to further evaluate the role of miR-301a and *RUNX3* in lung tumor cells. Introducing the miR-301a inhibitor, locked nucleic acid (LNA)-anti-miR-301a, led to upregulation of RUNX3 protein expression in A549 and H1299 cells (Fig. 5c and d). Furthermore, we found that miR-301a inhibition with 5 pmol and 10 pmol of LNA-anti-miR-301a at 48 h and 72 h significantly reduced the proliferation of A549 cells (Fig. 5e), but not H1299 cells (Fig. 5f). Yet concomitant knockdown of *RUNX3* did not reverse the reduced cellular proliferation mediated by miR-301a inhibition in A549 cells (Fig. 5g and h), indicating that other miR-301a target genes are involved in regulating cellular proliferation.

*PTEN* is a miR-301a target that suppresses cell and tumor growth in breast cancer [28], bladder cancer [29], and sarcoma [30]. *PTEN* mRNA level was significantly lower in 4 NSCLC cell lines than in 16HBE cells (Additional file 2: Figure S4a). PTEN protein levels was significantly upregulated by miR-301a inhibition in A549 cells, but not in H1299 cells (Additional file 2: Figure S4b). Knockdown of *PTEN* via small interfering RNA (siRNA) significantly enhanced proliferation of A549 cells when miR301a was inhibited (Additional file 2: Figure S4c and 4d). We next measured protein expression of Pten and two Ras effectors, phosphor-Akt and phosphor-Erk, in lung tumors from *Kras*^*LA2*^ and *miR-301a*^*−/−*^*;Kras*^*LA2*^ mice. We did not find significant changes in protein levels of Pten, phosphor-Akt and phosphor-Erk (Additional file 2: Figure S4e and 4f). miR-301a activates both NF-κB and Stat3 pro-inflammatory pathways to facilitate lung tumorigenesis [15] by targeting *Nkrf* and *Pias3*, respectively [12, 31]. We evaluated the expression of these two miR-301a target genes. However, Pias3 and Nkrf were down-regulated in lung tumors from *miR-301a*^*−/−*^*;Kras*^*LA2*^ mice compared with those in *Kras*^*LA2*^ mice (Additional file 2: Figure S4e). Overall, these data demonstrate that miR-301a deficiency did not upregulate the expression of its 3 target genes (*Pten*, *Pias3* and *Nkrf*) during *Kras*-driven lung tumorigenesis.

As *RUNX3* inhibits tumor cell metastasis through the regulation of the Wnt signaling pathway [18, 21], we sought to determine whether inhibition of miR-301a reduces cell migration by targeting *RUNX3*. We measured the migration of A549 and H1299 cells using a transwell migration assay. Cell migration was significantly reduced when LNA-anti-miR-301a was introduced into A549 and H1299 cells, whereas concomitant *RUNX3* knockdown partially reversed the migration phenotype (Fig. 5i and j). *β-catenin* expression was downregulated in both A549 and H1299 cells upon miR-301a inhibition (Fig. 5k). In addition, we also measured several extracellular matrix (ECM) associate proteins including fibronectin, vimentin, N-cadherin, E-cadherin MMP9 and MMP2 in both A549 and H1299. Our results shown that inhibition of miR-301a in A549 and H1299 significantly reduced N-cadherin and MMP9 expression but increased E-cadherin expression (Fig. 5l). Collectively, these data indicated that miR-301a may have a general impact in mesenchymal fate of lung cancer cells and provide strong evidence to support elevated *RUNX3* expression contributes to attenuated cellular migration in lung cancer cells with miR-301a inhibition and reduced lung tumorigenesis in miR-301a deficient mice.

### Deletion of miR-301a in mice inhibits tumor cell metastasis by elevating Runx3 and T cell responses

**Fig. 6:**
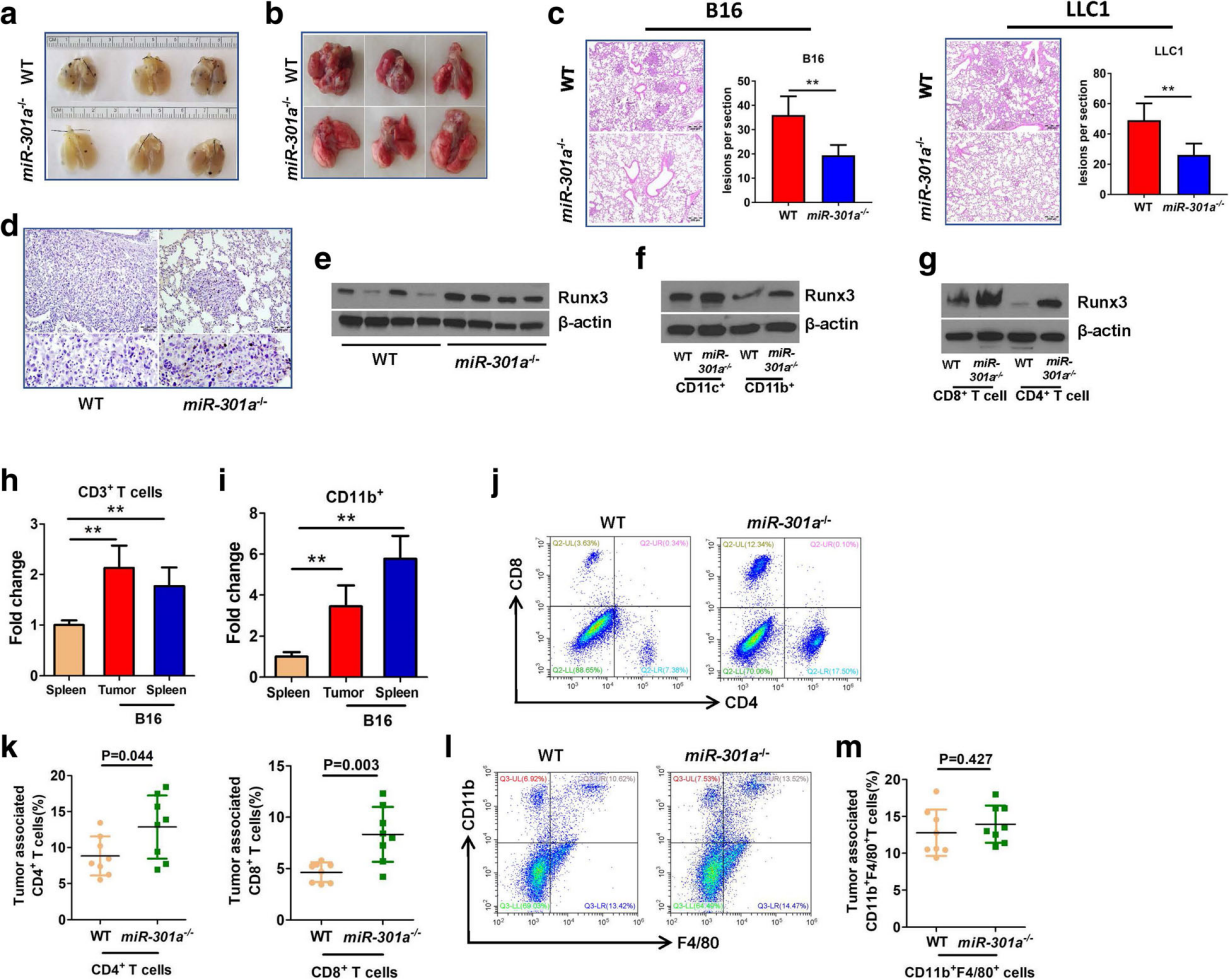
miR-301a deficiency in the tumor microenvironment inhibits tumor metastasis. Images of representative B16 tumors (**a**) and LLC1 (**b**) tumors developed in the lung of mice. WT and *miR-301a*^*−/−*^ mice (*n* = 8 per group) were implanted with B16 or LLC1 tumor cells by intravenous injection. (**c**) H&E-stained sections of lungs isolated from WT and KO mice implanted with B16 or LLC1 tumor cells. Scale bars = 200 μm. (**d**) Runx3 expression in lung tumor sections from WT and KO mice was determined by immunohistochemistry. Scale bars = 100 μm. (**e**) Western blot analysis of Runx3 expression in lung tumor tissues of WT (n = 4) and *miR-301a*^*−/−*^ (n = 4) mice. (**f**) CD11c^+^ and CD11b^+^ cells from lung tumors and (**g**) CD8^+^ and CD4^+^ cells from lung tumors of WT and *miR-301a*^*−/−*^ mice implanted with B16 tumor cells. Tumor-associated CD3^+^ cells (h) and CD11b^+^ cells (**i**) were purified from B16 tumors harvested from WT (*n* = 3) and *miR-301a*^*−/−*^ mice (n = 3). miR-301a expression was measured by qPCR. (**j**) Percentages of infiltrating CD8^+^ and CD4^+^ T cells isolated from B16 tumors from WT (n = 8) and *miR-301a*^*−/−*^ mice (n = 8) as analyzed by flow cytometry (pregated on CD3 events). (k) CD4^+^ and CD8^+^ T cells counts in lung tumor sections from WT (n = 8) and *miR-301a*^*−/−*^ mice (n = 8). (**l**) Percentages of infiltrating CD11b^+^ and F4/80^+^ cells isolated from B16 tumors developed in WT (n = 8) and *miR-301a*^*−/−*^ mice (n = 8) as analyzed by flow cytometry (pregated on CD3 events). (**m**) CD11b^+^ and F4/80^+^ T cells counts in lung tumor sections from WT (n = 8) and *miR-301a*^*−/−*^ mice (n = 8). Values represented the mean ± s.d. of three independent experiments. **P < 0.01 or *P < 0.05 indicates a significant difference between the indicated groups (one-way analysis of variance (ANOVA) in h and i and two-tailed, unpaired Student’s *t-*test in **k** and **m**)

**Fig. 7:**
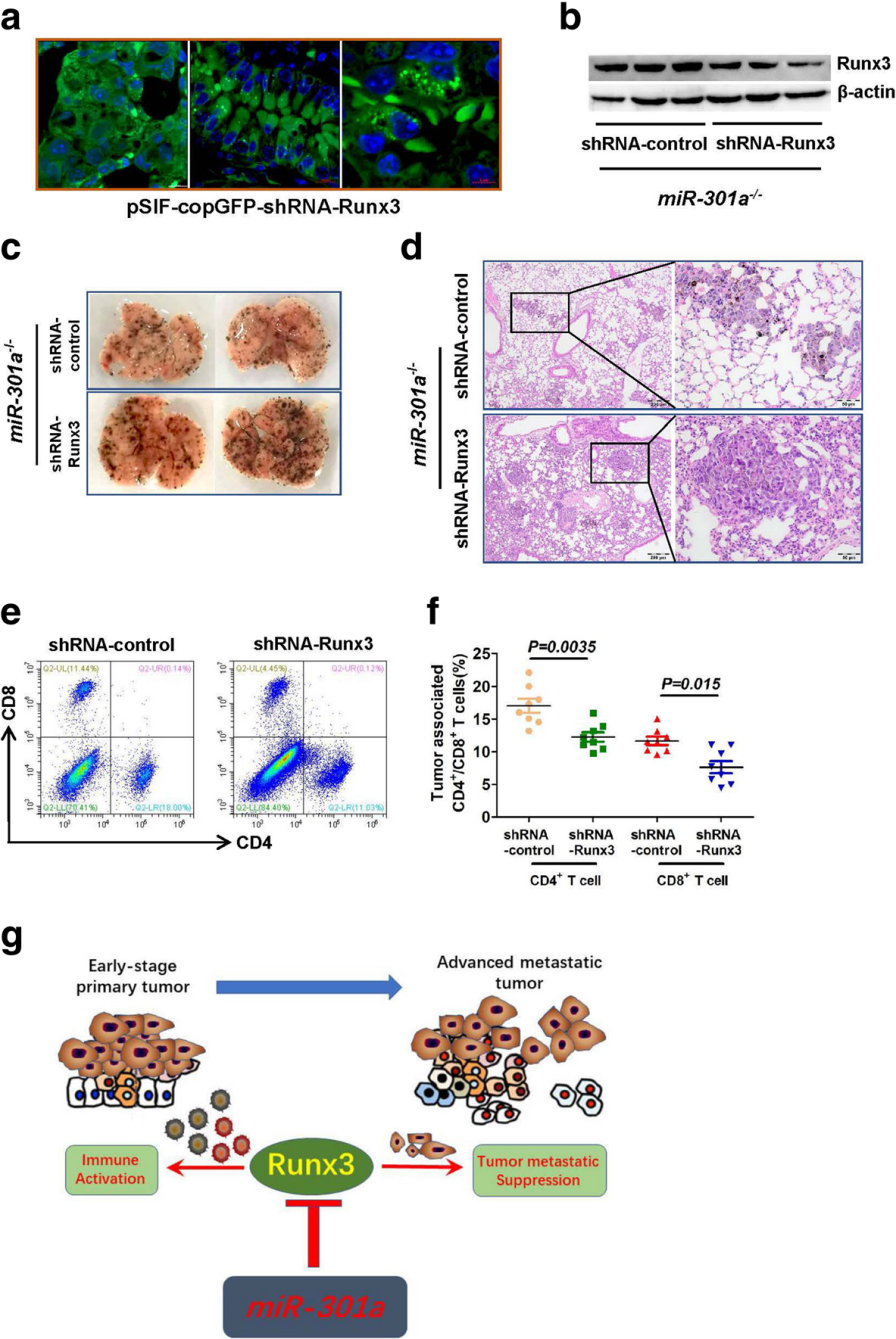
Inhibition of tumor metastasis in *miR-301a*^*−/−*^ mice correlates with elevated Runx3 expression and CD8^+^ T cell infiltration. *miR-301a*^*−/−*^ mice (n = 8 per group) were implanted with B16 tumor cells by intravenous injection. After 48 h, shRNA control or shRNA-Runx3 lentivirus was injected into *miR-301a*^*−/−*^ mice every day until mice were sacrificed and lung tissues collected. (**a**) Expression of GFP from the shRNA-Runx3 vector in B16 lung tumors. Scale bars = 10 μm or 5 μm. (**b**) Runx3 expression in lung sections from *miR-301a*^*−/−*^ mice (n = 3) with either shRNA-control or shRNA-Runx3 as determined by western blot. (**c**) Images of representative B16 tumors in the lung from *miR-301a*^*−/−*^ mice with either shRNA-control or shRNA-Runx3 lentivirus (n = 8 per group). (**d**) H&E-stained sections of lungs isolated from *miR-301a*^*−/−*^ mice (n = 8 per group) with B16 tumor cells. (**e**) Infiltrating T cells within lung tumors. Right panel: Percentages of infiltrating CD8^+^ and CD4^+^ T cells isolated from B16 tumors implanted in *miR-301a*^*−/−*^ mice with either shRNA-control or shRNA-Runx3 lentivirus as analyzed by flow cytometry (pregated on CD3 events). Left panel: CD4^+^ and CD8^+^ T cells counts in lung sections (n = 8 per group). (**f**) Schematic representation of the roles of miR-301a and *Runx3* in lung tumorigenesis. Values represented the mean ± s.d. of three independent experiments. **P < 0.01 or *P < 0.05 indicates a significant difference between the indicated groups (two-tailed, unpaired Student’s *t-*test in f)

To evaluate the function of miR-301a and *Runx3* in regulating tumor metastasis, we intravenously injected the melanoma cell line (B16) and Lewis lung carcinoma (LLC1) cells into WT and *miR-301a*^*−/−*^ mice. Lung tumors that developed from either B16 or LLC1 cells in *miR-301a*^*−/−*^ mice were significantly smaller than those in WT mice by visual inspection (Fig. 6a and b) and by H&E staining (Fig. 6c). Consistent with the results from *Kras* mutation mice, cell proliferation, but not apoptosis was significantly inhibited in B16 tumor from *miR-301a*^*−/−*^ mice compared with that in WT mice (Additional file 2: Figure S5a and 5b). We next examined Runx3 expression in metastatic tumors using immunostaining. *Runx3* was barely expressed in B16 tumors from WT mice, however, it was highly expressed in B16 tumors from *miR-301a*^*−/−*^ mice (Fig. 6d and e). To determine whether miR-301a deletion raises Runx3 expression in tumor-infiltrating immune cells, we isolated CD8^+^ T cells, CD4^+^ T cells, CD11b^+^ cells (including monocytes and macrophages), and CD11c^+^ cells (including dendritic cells), from B16 tumors developed in WT and *miR-301a*^*−/−*^ mice. We found significantly higher Runx3 expression in tumor-infiltrating immune cells isolated from B16 tumors from *miR-301a*^*−/−*^ mice compared to those from WT mice (Fig. 6f and g). Runx3 is a well-established regulator of CD4^+^ and CD8^+^ T cells [32, 33], so we assessed whether miR-301a deletion alters tumor stromal immune responses. When B16 cells were implanted into WT mice, miR-301a was highly expressed in both tumor-associated CD3^+^ T cells (Fig. 6h) and CD11b^+^ cells (Fig. 6i) compared with their counterparts in spleen of un-implanted WT mice. The proportion of tumor-associated CD4^+^ and CD8^+^ T cells as a percentage of all immune cells from B16 tumors isolated from *miR-301a*^*−/−*^ mice was higher than those from WT mice (Fig. 6j and k). There was no difference in the proportion of infiltrating macrophages (CD11b^+^F4/80^+^) between WT and *miR-301a*^*−/−*^ mice (Fig. 6l and m). These data support that attenuated tumor metastasis in *miR-301a*^*−/−*^ mice is related with elevated Runx3 expression in tumor-associated T cells. To determine whether increased Runx3 expression in *miR-301a*^*−/−*^ mice is responsible for recruiting T cells and reducing B16 tumor cell metastasis, we injected *miR-301a*^*−/−*^ mice intravenously with shRNA-Runx3 lentivirus after implantation with B16 tumor cells. Compared with the shRNA-control lentivirus, shRNA-Runx3 lentivirus significantly downregulated Runx3 expression in the tumors (Fig. 7a and b). Blockade of Runx3 expression sharply increased tumor metastasis (Fig. 7c and d), accompanied by fewer tumor-infiltrating CD4^+^ and CD8^+^ T cells (Fig. 7e). These data underscored the critical role of miR-301a in immune activation and tumor metastasis through Runx3 suppression during lung tumorigenesis (Fig. 7f).

## Discussion

In this study, we performed RNA-sequencing to identify the potential miR-301a targets and related signaling networks in *Kras*-induced lung tumorigenesis and determined the role of miR-301a in tumor cell proliferation and migration in NSCLC cell lines and in a syngeneic lung tumor metastasis model. Our major finding is that miR-301a deficiency reactivates the immune response in the tumor microenvironment by recruiting cytotoxic T cells through negatively regulating *Runx3* expression. Moreover, we show that miR-301a controls tumor metastasis by targeting *Runx3/β-catenin*, providing an underlying mechanism of miR-301a-mediated tumor promotion in NSCLC cells and during lung tumorigenesis.

miR-301a plays a dual role, oncogene-specific effect and pro-inflammatory mediator, in tumorigenesis [15, 34, 35]. Although loss of miR-301a in tumor microenvironment significantly reduces tumor growth, it is unclear which types of tumor-infiltrating immune cells are affected by aberrant expression of miR-301a. Previous studies have shown that miR-301a is markedly up-regulated after T cell activation [36] and miR-301a inhibition in CD4^+^ T cells reduces IL-17 secretion and modulated Th17 development [31], indicating the importance of miR-301a in Th17 cells. Our RNA-Sequencing data identify 49 genes related to CD8^+^ T cell activity within lung tumors developed in *Kras*-mutated mice with miR-301a knockout. We found, for the first time, that miR-301a controls CD8^+^ tumor-infiltrating lymphocytes in lung tumors. Deletion of miR-301a in tumor immune cells increases the amounts of tumor-infiltrating CD8^+^ T cells, resulting in attenuated tumor growth and reduced metastasis. Given that activation of *Kras* in the mouse lung generates an inflammatory process [37], it is likely that the reduced tumor development in miR-301a-deficient mice is due to a limited pro-inflammatory response caused by reactivated immune phenotypic changes. Consistent with that overexpression of miR-301a decreases IFN-γ release from antigen-specific cytotoxic T cells [14], our experiments show that IFN-γ release is significantly upregulated in *Kras*-driven lung tumors upon miR-301a deletion. Such altered IFN-γ expression is observed in neither WT nor *miR-301a*^*−/−*^ mice without *Kras* mutation. These data indicate that miR-301a deletion results in enhanced recruitment of CD8^+^ T cells, which are responsible for increased production of tumoricidal IFN-γ. In addition to IFN-γ, several other pro-inflammatory cytokines including IL-6, IL-1α, and IL-1β are down-regulated in m*iR301a*^*−/−*^*;Kras*^*LA2*^ lung tumors, like due to that miR-301a deletion reduces the activation of NF-κB and Stat3 [12, 31].

Our studies identify *RUNX3* as a novel target gene of miR-301a in NSCLC cell lines and *Kras* mutated mice. *RUNX3,* as a tumor suppressor gene, is frequently inactivated in human cancer cell lines and patient samples [38, 39]. Loss of *RUNX3* occurs more frequently in invasive lung adenocarcinoma than in pre-invasive lesions [40]. Targeted inactivation of *Runx3* in mouse lung results in lung adenoma and accelerates *Kras*-induced development of lung adenocarcinoma as well as loss of *Trp53* function, indicating that loss of *RUNX3* is a crucial early event in lung tumorigenesis [24]. As a regulator of T cell development, *Runx3* increases the amounts of tumor-infiltrating CD8^+^ T cells and restrains tumor growth [32]. Our results from *Kras* transgenic mice and B16/LLC1 syngeneic xenografts show that *Runx3* is significantly upregulated in miR-301a-deficient lung tumors. Down-regulation of *Runx3* by shRNA in B16 metastatic tumors grown in *miR-301a*^*−/−*^ mice increases the amounts of tumor-infiltrating CD8^+^ T cells and reduces lung metastasis. Interestingly, down-regulation of *PTEN*, but not that of *RUNX3*, reverses the reduced cell proliferation of NSCLC cell lines mediated by miR-301a inhibition.

## Conclusions

Overall, our results show that miR-301a promotes lung tumorigenesis via *RUNX3* directly involving tumor cell metastasis and the tumor immune response events. Loss of miR-301a in lung tumor cells inhibits tumor metastasis by targeting *RUNX3*, whereas deletion of miR-301a in tumor microenvironment reduces the tumorigenesis by elevating CD8^+^ cytotoxic T cells by negatively regulating *RUNX3* (Fig. 7g). This is the first demonstration that miR-301a deficiency enhances CD8^+^ T cell accumulation by negatively regulating Runx3 that are implicated in the anti-tumor immunity.

## Additional files

Additional file 1: **Table S1.** Functional annotation clustering for GO (gene ontology) terms involving DEGs in lung tissue between *Kras*^*LA2*^ mice and *miR301a*^*−/−*^*;Kras*^*LA2*^ mice. **Table S2.** Functional annotation clustering for GO terms involving up and down regulated genes in lung tissue between *Kras*^*LA2*^ mice and *miR301a*^*−/−*^*;Kras*^*LA2*^ mice. **Table S3.** The canonical pathways identified between *Kras*^*LA2*^ and *miR-301a*^*−/−*^*;Kras*^*LA2*^. **Table S4.** 283 molecules related with lung tumors were identified between *Kras*^*LA2*^ and *miR-301a*^*−/−*^*;Kras*^*LA2*^ mice. **Table S5.** 47 molecules related with CD8^+^ T lymphocyte were identified between *Kras*^*LA2*^ and *miR-301a*^*−/−*^*;Kras*^*LA2*^ mice. **Table S6.** The primer used for real-time PCR assay. (DOCX 103 kb)

Additional file 2: **Figure S1.** IPA Interaction networks analysis of significant DEGs in lung tissue between *Kras*^*LA2*^ and *miR-301a*^*−/−*^*Kras*^*LA2*^ mice and identified IFNG and CTNNB1 as the central node in the top regulated network. **Figure S2.** Runx3 is a direct target of miR-301a. **Figure S3.** The mRNA and protein expression of 5 genes in NSCLC cell lines. **Figure S4.** The effects of PTEN on cell proliferation in A549 and lung tumorigenesis. **Figure S5.** The effects of miR-301a on cell proliferation and apoptosis in mouse xenografts (DOCX 4049 kb)

## Abbreviations

EMT: epithelial-to-mesenchymal transition
FDR: false discovery rate
H&E: hematoxylin and eosin
IPA: Ingenuity Pathway Analysis
LNA: locked nucleic acid
MEFs: mouse embryonic fibroblast
RPKM: reads per kilobase of exon per million mapped reads
RUNX3: transcription factor 3
TGF-β: transforming growth factor-β
WT: wild-type
