# Supplementary material for: miR-301a promotes lung tumorigenesis by suppressing Runx3
Source: Mol Cancer. 2019 May 23;18:99. doi: 10.1186/s12943-019-1024-0 (PMC6532219; doi:10.1186/s12943-019-1024-0)
Supplement: Supplementary file 1 — Table S1. Functional annotation clustering for GO (gene ontology) terms involving DEGs in lung tissue between KrasLA2 mice and miR301a−/−;KrasLA2 mice. Table S2. Functional annotation clustering for GO terms involving up and down regulated genes in lung tissue between KrasLA2 mice and miR301a−/−;KrasLA2 mice. Table S3. The canonical pathways identified between KrasLA2 and miR-301a−/−;KrasLA2. Table S4. 283 molecules related with lung tumors were identified between KrasLA2 and miR-301a−/−;KrasLA2 mice. Table S5. 47 molecules related with CD8+ T lymphocyte were identified between KrasLA2 and miR-301a−/−;KrasLA2 mice. Table S6. The primer used for real-time PCR assay. (DOCX 103 kb) [file 12943_2019_1024_MOESM1_ESM.docx]

**Supplementary Tables for**

***Xun Li* et al, “miR-301a Promotes Lung Tumorigenesis by Suppressing Runx3”**

Table of Contents

**Supplementary Table 1.** Functional annotation clustering for GO (gene ontology) terms involving DEGs in lung tissue between *Kras^LA2^* mice and *miR301a^-/-^;Kras^LA2^* mice. **……………2**

**Supplementary Table 2.** Functional annotation clustering for GO terms involving up and down regulated genes in lung tissue between *Kras^LA2^* mice and *miR301a^-/-^;Kras^LA2^* mice. **……………5**

**Supplementary Table 3.** The canonical pathways identified between *Kras^LA2^* and *miR-301a^-/-^;Kras^LA2^*. **…………………....………………………………………………………………… 7**

**Supplementary Table 4.** 283 molecules related with lung tumors were identified between *Kras^LA2^* and *miR-301a^-/-^;Kras^LA2^* mice.  **…………………………………………………………...... 16**

**Supplementary Table 5.** 47 molecules related with CD8^+^ T lymphocyte were identified between *Kras^LA2^* and *miR-301a^-/-^;Kras^LA2^* mice.  **……………………………………………………... 25**

**Supplementary Table 6.** The primer used for real-time PCR assay. **…………………….. 27**

**Supplementary Table 1** Functional annotation clustering for GO (gene ontology) terms involving DEGs in lung tissue between *Kras^LA2^* mice and *miR301a^-/-^;Kras^LA2^* mice (P-value<0.05, Benjamini<0.05).

| Term | Count | % | P-value | Fold Enrichment | Benjamini | Genes |
| --- | --- | --- | --- | --- | --- | --- |
| GO:0002376~  immune system process | 87 | 5.534351 | 9.22E-20 | 2.91304928 | 4.18E-16 | ZC3HAV1, CD8A, LY86, TLR1, PTPN22, HP, LY9, SKAP1, C1QC, TLR9, ISG20, CFP, H60B, NLRC5, LBP, CSK, CFD, MX1, SYK, ICOSL, LYN, LY96, BPIFA1, CD3E, PIK3CD, PRG2, HERC6, CD40, FCAMR, PRKCB, PRKCQ, C1QB, LAT2, CD86, CAMK4, LAX1, SERPINA3G, SLPI, LRMP, CD300LF, PTMS, GPR183, HMGB2, BPIFB1, FCNA, PML, KLRK1, OAS3, UNC93B1, RSAD2, OAS2, SP110, SEC14L1, CD74, SERINC3, SERINC5, PTK2B, TAP1, SFTPD, IIGP1, CD4, CSF1R, TLR11, ITK, CD8B1, CR2, LGALS3, OLR1, THEMIS, HC, TNFRSF13B, TNFRSF13C, ANXA1, SLAMF6, H2-AB1, CD5L, H2-Q7, AIM2, CD180, BTLA, LCN2, CD55, H2-EB1, CD79B, CD79A, THEMIS2, CD14 |
| GO:0007049~cell cycle | 96 | 6.10687 | 6.71E-11 | 2.00507312 | 1.52E-07 | KIF23, E2F2, E2F4, E2F8, KNTC1, MLH1, CUZD1, CCNE1, MCM8, EVI5, CASP8AP2, OIP5, CCSAP, PAK4, INCENP, H2AFX, CCNA2, ASPM, CCAR1, CDCA3, KIF2A, ESCO1, KIF11, LIG1, SGOL1, RBL1, TPX2, PIM1, NUSAP1, MCM2, UBE2C, MCM3, MCM4, MCM5, NCAPD3, ESCO2, RAD50, MCM6, NCAPD2, RBBP8, CCND1, UHRF1, MAD2L1, CCND3, SYCP3, RIF1, MAPK3, RGCC, RBM38, PSME3, HAUS3, MPLKIP, TICRR, GNAI1, LIN9, BEX2, NEDD9, ANLN, CHEK1, NCAPH, NIPBL, GADD45GIP1, NCAPG2, TFDP2, BUB1, NPAT, USP37, FBXO5, ZWILCH, TERF2, TFDP1, CKAP2, TRP53, PARD6B, SETDB2, MKI67, IKZF1, NASP, HACE1, BRCA2, CENPE, NDC80, SMC2, SMC3, BRCA1, SMC4, CCNB1, FAM64A, CDKN1A, CCNB2, DUSP1, KIF20B, CUL4B, MIS18BP1, CIT, CCNDBP1 |
| GO:0002250~adaptive immune response | 33 | 2.099237 | 2.00E-08 | 3.0445737 | 3.02E-05 | GPR183, CD8A, KLRK1, UNC93B1, LY9, SKAP1, CD74, PTK2B, TAP1, CD4, CSK, SYK, ITK, ICOSL, CD8B1, LYN, THEMIS, PIK3CD, TNFRSF13B, SLAMF6, TNFRSF13C, ANXA1, CTSS, FCAMR, PRKCB, BTLA, LAT2, CD86, CAMK4, LAX1, SERPINA3G, CD79B, CD79A |
| GO:0042113~B cell activation | 14 | 0.890585 | 7.68E-08 | 6.19095133 | 8.71E-05 | GAPT, ICOSL, IKZF3, CR2, PIK3CD, CD40, PRKCB, CD86, LAT2, LAX1, MS4A1, CD79A, BANK1, BLNK |
| GO:0007155~cell adhesion | 73 | 4.643766 | 7.94E-08 | 1.93022739 | 7.21E-05 | ATP1B1, ATP1B2, NPNT, BCAM, CUZD1, LY9, CTNNB1, CD96, ICAM1, F11R, PTPRF, EGFL7, ICAM2, THY1, LAMC2, MFAP4, EMP2, CD226, PARVB, ADAM15, ITGAL, SIGLECE, ACHE, CYP1B1, CTNND2, NEDD9, CDH1, EPHB4, CD24A, CDH8, VCAM1, CD9, SORBS3, LPXN, LAMB3, LAMB2, GLYCAM1, ITGAX, KLRA8, SORBS2, PTK2B, COL6A2, CD2, KLRA4, COL6A1, CD22, GP1BA, CD4, GPNMB, FN1, SPP1, OLR1, SELL, ADAM23, ITGA1, NID2, ITGA4, STAB2, PCDH17, CD63, MCAM, KITL, VWF, LYVE1, COL14A1, STAB1, LAMA5, ITGA8, SULF1, PKP4, CYFIP2, PERP, CDH11 |
| GO:0006260~DNA replication | 28 | 1.78117 | 6.91E-07 | 2.91931038 | 5.23E-04 | BLM, TICRR, POLA1, PRIM1, MCM8, PRIM2, ORC4, ORC6, ORC2, SSRP1, CCDC88A, DTL, NASP, LIG1, FAM111A, POLE, WRN, MCM2, MCM3, RMI1, MCM4, BRCA1, MCM5, MCM6, RRM2, RRM1, PCNA, NFIC |
| GO:0030890~positive regulation of B cell proliferation | 15 | 0.954198 | 2.59E-06 | 4.47352796 | 0.001680931 | MEF2C, GPR183, PTPRC, BST1, TNFRSF13C, ATAD5, CD40, CD74, CD38, CDKN1A, TFRC, CLCF1, BCL2, TCF3, SASH3 |
| GO:0006955~immune response | 45 | 2.862595 | 2.80E-06 | 2.12163642 | 0.00158967 | ENPP2, TLR1, CCL9, CXCL9, OAS3, OAS2, CD24A, FTH1, CD74, CCL6, TLR9, CXCL10, CD4, LTB, BLNK, FYB, TLR11, SMAD6, PRG2, H2-AB1, CTSS, GM13304, WAS, FCAMR, VAV1, H2-DMB2, CCL17, CCR9, SERPINB9, CCR7, CCR6, PPBP, H2-OA, H2-EB2, LAX1, CXCL13, CXCL15, H2-EB1, H2-OB, IRF8, SLPI, TGTP2, NGFR, IRAK1BP1, LCP2 |
| GO:0045087~innate immune response | 59 | 3.753181 | 3.09E-06 | 1.89155674 | 0.001556126 | ZC3HAV1, LY86, TLR1, LY9, C1QC, TLR9, ISG20, CFP, NLRC5, ANG, LBP, CSK, MX1, CFD, SYK, LYN, LY96, BPIFA1, C4B, CAMP, PIK3CD, HERC6, C1QB, SLPI, ADAM15, BPIFB1, HMGB2, FCNA, BLK, PML, KLRK1, OAS3, UNC93B1, RSAD2, SP110, OAS2, TRIM10, SEC14L1, SERINC3, SERINC5, REL, PTK2B, SFTPD, IIGP1, CSF1R, TYROBP, ITK, TLR11, CR2, LGALS3, HC, SLAMF6, ANXA1, CD180, AIM2, LCN2, CD55, CLEC7A, CD14 |
| GO:0032496~response to lipopolysaccharide | 36 | 2.290076 | 3.43E-06 | 2.34349282 | 0.001556349 | ALDOA, OTUD5, HMGB2, SNCA, CXCL9, PTPN22, SCGB1A1, GCH1, CXCL10, VCAM1, SLC11A1, CD96, CASP9, IL10RA, CASP8, CNR2, NKX2-1, LBP, CASP1, TCF3, LY96, ELANE, SPARC, CD40, STAT1, PCK1, CCR7, PPBP, CXCL13, JUN, CXCL15, MAPK3, PLCG2, SLPI, MPO, NGFR |
| GO:0042542~response to hydrogen peroxide | 17 | 1.081425 | 5.87E-06 | 3.75879188 | 0.002418072 | STAR, CRYAB, LIG1, STK26, HP, STAT1, MMP2, SDC1, DUSP1, PTK2B, JUN, BCL2, HMOX1, CAT, AREG, SLC4A1, MB |
| GO:0030217~T cell differentiation | 14 | 0.890585 | 8.22E-06 | 4.37896558 | 0.003104958 | PTPRC, BCL2A1D, IKZF1, LEPR, BCL2, BCL11A, PTPN22, CD4, IL7R, VAV1, NFATC3, CTNNB1, GIMAP1, RHOH |
| GO:0008630~intrinsic apoptotic signaling pathway in response to DNA damage | 16 | 1.017812 | 1.04E-05 | 3.79973733 | 0.003630578 | IER3, CRIP1, PML, BRCA2, PRKDC, MLH1, MBD4, BRCA1, EI24, BCL2A1D, BCL2A1B, CASP9, BCL2A1A, BCL2, HMOX1, MYC |
| GO:0006974~cellular response to DNA damage stimulus | 59 | 3.753181 | 1.40E-05 | 1.80148261 | 0.004518023 | ERCC6L2, MORF4L2, MLH1, RPS27L, SETX, FANCL, FANCM, MCM8, CASP9, H2AFX, TOP2A, MYC, SGK1, REV1, LYN, GEN1, DTL, LIG1, USP1, NEIL3, POLE, MBD4, TOPBP1, STXBP4, RAD50, GTF2H2, RBBP8, WDR48, CCND1, UHRF1, RFWD3, RIF1, MAPK3, BLM, TICRR, PRKDC, CHEK1, NIPBL, BCL2, WAC, NTHL1, TRP53, SSRP1, BOD1L, NFRKB, BRIP1, BRCA2, SMC6, ATAD5, WRN, SMC3, BRCA1, CDKN1A, CUL4A, PARP9, PARPBP, PCNA, CUL4B, PARP1 |
| GO:0051607~defense response to virus | 30 | 1.908397 | 3.57E-05 | 2.30373296 | 0.010731415 | RNASEL, APOBEC1, CD8A, ZC3HAV1, SLFN9, CXCL9, UNC93B1, OAS3, PML, SLFN8, RSAD2, OAS2, TLR9, CXCL10, ISG20, SERINC3, NLRC5, SERINC5, ITGAX, ISG15, BCL2, MX1, NCBP3, PTPRC, TRIM34A, CD40, NCR1, STAT2, ABCC9, CD86 |
| GO:0042493~response to drug | 49 | 3.117048 | 3.95E-05 | 1.85363292 | 0.011140949 | TSPO, APOBEC1, STAR, SNCA, MMP2, SCGB1A1, CTNNB1, CAT, RPN2, CASP1, HADH, PMS1, ICAM1, HSP90AA1, FECH, MYO6, LYN, GSTT1, POR, PRKCB, CD38, CD86, CCND1, JUN, PEBP1, HMGB2, CDH1, GCLM, ARG1, TYMS, PLIN2, PTK2B, BCL2, ABCD3, TCF3, SREBF1, TRP53, LPL, LGALS1, ANXA1, ATP1A1, STAT1, LCN2, CCNB1, HDAC4, EI24, CDKN1A, DNMT1, IGFBP2 |
| GO:0097421~liver regeneration | 11 | 0.699746 | 4.04E-05 | 4.8643189 | 0.010740362 | EGFR, TYMS, SRSF5, CCND1, RPS16, UCP2, HMOX1, EZH2, RAP1A, MYC, REG1 |
| GO:0006270~DNA replication initiation | 10 | 0.636132 | 4.60E-05 | 5.34338061 | 0.011537765 | CCNE1, POLA1, ORC4, TOPBP1, ORC6, MCM2, MCM3, MCM4, MCM5, MCM6 |
| GO:0050853~B cell receptor signaling pathway | 15 | 0.954198 | 5.94E-05 | 3.49748549 | 0.014092832 | MEF2C, PTPN6, PTPRC, LYN, BLK, PRKCB, CD38, LAT2, KLHL6, CD19, BCL2, PLCG2, CD79B, CD79A, SYK |
| GO:0051301~cell division | 52 | 3.307888 | 6.24E-05 | 1.78303182 | 0.014052057 | KIF23, KNTC1, CUZD1, CCNE1, EVI5, OIP5, INCENP, CCSAP, CCNA2, ASPM, CDCA3, KIF2A, KIF14, KIF11, LIG1, SGOL1, TPX2, NUSAP1, UBE2C, NCAPD3, MCM5, RBBP8, NCAPD2, CCND1, MAD2L1, SYCP3, CCND3, HAUS3, MPLKIP, GNAI1, NEDD9, ANLN, NCAPH, NCAPG2, BUB1, USP37, FBXO5, ZWILCH, PARD6B, SETDB2, NDC80, CENPE, CDC27, SMC2, SMC3, SMC4, CCNB1, FAM64A, CCNB2, KIF20B, MIS18BP1, CIT |
| GO:0007568~aging | 30 | 1.908397 | 6.92E-05 | 2.22383471 | 0.014853169 | TSPO, SNCA, PAX5, GCLM, TIMP1, ATP2B1, VCAM1, ARG1, TYMS, CASP9, GSN, DMD, RPN2, CAT, CIITA, SREBF1, NOX4, CRYAB, PCK1, PPP1R9A, CD86, LRP1, UCP2, JUN, EIF2S1, MPO, PEBP1, CTSC, IGFBP2, SCP2 |
| GO:0042102~positive regulation of T cell proliferation | 16 | 1.017812 | 9.16E-05 | 3.20602837 | 0.018722805 | ITGAL, PTPRC, CD3E, AIF1, ANXA1, TNFRSF13C, VCAM1, PRKCQ, CD86, CORO1A, CCR7, CD80, TFRC, CD4, SLC4A1, SASH3 |
| GO:0050852~T cell receptor signaling pathway | 14 | 0.890585 | 1.07E-04 | 3.52034488 | 0.020827723 | FYB, ITK, PTPRC, BCL2A1D, CD3E, THEMIS, PLCG2, PTPN22, SKAP1, THEMIS2, CLEC2I, DENND1B, THY1, LCP2 |
| GO:0007067~mitotic nuclear division | 41 | 2.608142 | 1.10E-04 | 1.89815398 | 0.020637047 | KIF23, HAUS3, MPLKIP, KNTC1, NEDD9, ANLN, NCAPH, OIP5, NCAPG2, INCENP, CCSAP, BUB1, FBXO5, USP37, ZWILCH, CCNA2, ASPM, KIF2A, CDCA3, SETDB2, KIF11, SGOL1, TPX2, NUSAP1, CENPE, NDC80, UBE2C, SMC2, SMC3, NCAPD3, SMC4, RBBP8, NCAPD2, CCNB1, FAM64A, CCNB2, MAD2L1, SYCP3, KIF20B, MIS18BP1, CIT |
| GO:0006954~inflammatory response | 48 | 3.053435 | 1.15E-04 | 1.78941118 | 0.02064075 | HMGB2, TSPAN2, NDST1, AIF1, LY86, TLR1, CCL9, CXCL9, CCL6, TLR9, CXCL10, SLC11A1, REL, CYP26B1, CNR2, REG3G, CSF1R, SPP1, CIITA, TLR11, LYN, OLR1, HC, C4B, LY96, PIK3CD, ANXA1, CHST2, CD5L, GM13304, CD40, CELA1, AGER, CD180, AIM2, CCL17, PRKCQ, SDC1, CCR7, CAMK4, CXCL13, STAB1, CCR3, CXCL15, CLEC7A, NGFR, THEMIS2, CD14 |
| GO:0051726~regulation of cell cycle | 22 | 1.399491 | 1.35E-04 | 2.51902229 | 0.023343221 | TRP53, E2F2, PTPRC, SGK1, E2F4, DTL, FIGNL1, RBL1, SIPA1, SKP2, TACC3, CCNE1, CCND1, CDKN1A, SRSF5, CCND3, JUN, BCL2, RGCC, TSC2, NPM1, CCNDBP1 |
| GO:0006281~DNA repair | 45 | 2.862595 | 1.40E-04 | 1.81473304 | 0.023286723 | ERCC6L2, BLM, TICRR, MORF4L2, MLH1, PRKDC, RPS27L, CHEK1, ANKLE1, SETX, FANCL, FANCM, MCM8, NPM1, H2AFX, NTHL1, SSRP1, BOD1L, REV1, GEN1, NEIL3, LIG1, NFRKB, USP1, POLE, BRCA2, BRIP1, SMC6, TOPBP1, MBD4, WRN, GTF2H2, BRCA1, RAD50, SMC3, RBBP8, UHRF1, RFWD3, CUL4A, PARP9, PARPBP, PCNA, CUL4B, PARP1, USP45 |
| GO:0007076~mitotic chromosome condensation | 7 | 0.445293 | 2.35E-04 | 6.90529187 | 0.037453146 | NCAPH, NCAPG, NUSAP1, SMC2, NCAPD3, SMC4, NCAPD2 |
| GO:0030097~hemopoiesis | 18 | 1.145038 | 2.98E-04 | 2.68411677 | 0.045596615 | IKZF1, LYN, EPAS1, CRIP2, BRCA2, SOX6, TACC3, CTNNB1, TAL1, CUL4A, BCL2, CXCL15, TXNRD2, ANGPT1, RUNX1, RUNX3, ADD2, CSF1R |
| GO:0007159~leukocyte cell-cell adhesion | 9 | 0.572519 | 3.16E-04 | 4.80904255 | 0.046664847 | VCAM1, ITGAL, ICAM1, PTPRC, OLR1, ROCK1, ITGA4, CD24A, SYK |
| GO:0007569~cell aging | 10 | 0.636132 | 3.28E-04 | 4.27470449 | 0.046955332 | NOX4, TRP53, ICAM1, TBX3, BCL2, NPM1, PML, BRCA2, WRN, PDCD4 |
| GO:0042832~defense response to protozoan | 10 | 0.636132 | 3.28E-04 | 4.27470449 | 0.046955332 | SLC11A1, CD37, TSPAN32, GBP9, IRF8, GBP10, IIGP1, IRF4, CD40, GBP3 |

**Supplementary Table 2** Functional annotation clustering for GO terms involving up and down regulated genes in lung tissue between *Kras^LA2^* mice and *miR301a^-/-^;Kras^LA2^* mice.

| Category Term | GO ID | Statistics |
| --- | --- | --- |
| *Up-regulated* |  |  |
| Immune system process | GO:0002376 | C:71;%:0.046;P:2.08E-19;B:7.12E-16;FDR:3.80E-16 |
| Cell cycle | GO:0007049 | C:88;%:0.057;P:2.58E-16;B:3.81E-13;FDR:4.11E-13 |
| Adaptive immune response | GO:0002250 | C:31;%:0.02;P:7.59E-11;B:8.67E-08;FDR:1.39E-07 |
| B cell activation | GO:0042113 | C:14;%:0.009;P:1.11E-09;B:9.53E-07;FDR:2.03E-06 |
| DNA replication | GO:0006260 | C:27;%:0.018;P:2.22E-09;B:1.52E-06;FDR:4.06E-06 |
| Cell division | GO:0051301 | C:49;%:0.032;P:3.53E-08;B:2.02E-05;FDR:6.46E-05 |
| Mitotic nuclear division | GO:0008285 | C:40;%:0.026;P:5.82E-08;B:2.85E-05;FDR:1.06E-04 |
| Cellular response to DNA damage stimulus | GO:0006974 | C:52;%:0.034;P:8.11E-08;B:3.47E-05;FDR:1.48E-04 |
| Defense response to virus | GO:0051607 | C:29;%:0.019;P:1.13E-07;B:4.30E-05;FDR:2.06E-04 |
| DNA repair | GO:0006281 | C:42;%:0.027;P:3.10E-07;B:1.06E-04;FDR:5.66E-04 |
| B cell receptor signaling pathway | GO:0050853 | C:15;%:0.01;P:1.06E-06;B:3.31E-04;FDR:1.94E-03 |
| Positive regulation of B cell proliferation | GO:0030890 | C:13;%:0.008;P:2.17E-06;B:6.20E-04;FDR:3.97E-03 |
|  |  |  |
| *Down-regulated* |  |  |
| Cell adhesion | GO:0007155 | C:35;%:7.72;P:9.25E-09;B:2.19E-05;FDR:1.62E-05 |
| Response to drug | GO:0042493 | C:25;%:5.52;P:1.25E-06;B:1.48E-03;FDR:2.20E-03 |
| Extracellular matrix organization | GO:0030198 | C:13;%:2.87;P:1.28E-05;B:1.00E-02;FDR:2.24E-02 |
| Epithelial tube branching involved in lung morphogenesis | GO:0060441 | C:7;%:1.55;P:1.37E-05;B:8.08E-03;FDR:2.41E-02 |
| Epithelial cell differentiation | GO:0030855 | C:10;%:2.2;P:2.22E-05;B:1.04E-02;FDR:3.88E-02 |
| Cell-cell adhesion | GO:0098609 | C:16;%:3.53;P:3.41E-05;B:1.33E-02;FDR:5.97E-02 |
| Wound healing, spreading of epidermal cells | GO:0035313 | C:5;%:1.1;P:1.19E-04;B:3.94E-02;FDR:2.09E-01 |
| Angiogenesis | GO:0001525 | C:17;%:3.75;P:1.44E-04;B:4.17E-02;FDR:2.52E-01 |
| Cellular response to tumor necrosis factor | GO:0071356 | C:11;%:2.43;P:2.30E-04;B:5.86E-02;FDR:4.03E-01 |
| Multicellular organismal water homeostasis | GO:0050891 | C:4;%:0.88;P:3.98E-04;B:8.97E-02;FDR:6.95E-01 |
| Response to hypoxia | GO:0001666 | C:14;%:3.09;P:5.33E-04;B:1.08E-01;FDR:9.30E-01 |
| Negative regulation of endothelial cell proliferation | GO:0001937 | C:6;%:1.32;P:5.64E-04;B:1.05E-01;FDR:9.84E-01 |
| Lung development | GO:0030324 | C:11;%:2.43;P:6.02E-04;B:1.04E-01;FDR:1.05E+00 |

Note: C, the number of reference genes in the category; %, the percentage of reference genes; P, the P value; B, Benjamini value; FDR, the False Discovery Rate

**Supplementary Table 3** The canonical pathways identified between *Kras^LA2^* and *miR-301a^-/-^;Kras^LA2^* (-logP >1.3 ).

| Ingenuity Canonical Pathways | -log(p-value) | Ratio | z-score | Molecules |
| --- | --- | --- | --- | --- |
| B Cell Development | 9.73 | 0.636 | #NUM! | HLA-DOA,CD19,CD79B,HLA-A,CD79A,HLA-DQB1,IL7R,PTPRC,CD40,CD80,HLA-DMB,CD86,HLA-DOB,HLA-DRB5 |
| Primary Immunodeficiency Signaling | 7.14 | 0.452 | #NUM! | BLNK,IL2RG,CD19,CD3E,CD4,CIITA,CD79A,TAP1,TNFRSF13C,IL7R,PTPRC,CD40,ICOS,TNFRSF13B |
| Cell Cycle Control of Chromosomal Replication | 6.84 | 0.346 | #NUM! | MCM5,LIG1,MCM6,ORC2,MCM8,POLA1,POLE,ORC6,MCM4,MCM3,PCNA,CDK8,PRIM1,MCM2,CDK17,TOP2A,PRIM2,ORC4 |
| iCOS-iCOSL Signaling in T Helper Cells | 6.81 | 0.257 | 4.2 | HLA-DOA,CAMK4,CD3E,HLA-A,NFATC3,NFKBIE,CD4,HLA-DQB1,PTPRC,HLA-DMB,ICOSLG/LOC102723996,ITK,IL2RB,IL2RG,PRKCQ,CSK,FGFR2,TLR9,CD40,CD80,GRAP2,ICOS,HLA-DOB,PIK3CD,VAV1,HLA-DRB5,LCP2 |
| CD28 Signaling in T Helper Cells | 6.67 | 0.248 | 2.683 | HLA-DOA,CAMK4,CD3E,HLA-A,NFATC3,NFKBIE,CD4,HLA-DQB1,PTPRC,JUN,ACTR3,HLA-DMB,ITK,PTPN6,PRKCQ,CSK,FGFR2,TLR9,CD80,WAS,GRAP2,SYK,CD86,HLA-DOB,PIK3CD,VAV1,HLA-DRB5,LCP2 |
| Th1 Pathway | 6.63 | 0.252 | 4.264 | HLA-DOA,ICAM1,CD3E,HLA-A,KLRD1,NFATC3,CD4,HLA-DQB1,HLA-DMB,IL27RA,STAT1,ICOSLG/LOC102723996,KLRC1,RUNX3,PRKCQ,FGFR2,TLR9,STAT4,CD80,CD40,ICOS,IL10RA,CD86,HLA-DOB,PIK3CD,VAV1,HLA-DRB5 |
| Th1 and Th2 Activation Pathway | 6.29 | 0.217 | #NUM! | CCR3,HLA-DOA,ICAM1,CD3E,HLA-A,NFATC3,KLRD1,CD4,HLA-DQB1,JUN,HLA-DMB,IL27RA,STAT1,TIMD4,ICOSLG/LOC102723996,KLRC1,IL2RB,RUNX3,IL2RG,PRKCQ,IKZF1,FGFR2,TLR9,STAT4,CD40,CD80,ICOS,IL10RA,CD86,HLA-DOB,VAV1,PIK3CD,HLA-DRB5 |
| Communication between Innate and Adaptive Immune Cells | 6.23 | 0.306 | #NUM! | Tlr11,CD79B,HLA-A,CD4,CD79A,CD83,TLR9,TNFRSF13C,CD8B,Ccl9,CXCL10,HLA-G,CD80,CD40,TLR1,CD86,TNFRSF13B,CCR7,HLA-DRB5 |
| B Cell Receptor Signaling | 5.96 | 0.207 | 3.157 | BLNK,RAC2,CAMK4,PTK2B,POU2F2,NFATC3,NFKBIE,PTPRC,PAX5,JUN,MAPK3,CD22,CD19,PTPN6,PRKCQ,APBB1IP,CD79B,FCGR2A,CSK,FGFR2,CD79A,TLR9,TCF3,RAP1A,EBF1,DAPP1,PLCG2,SYK,LYN,MEF2C,PIK3CD,VAV1,BCL2A1,PRKCB |
| Th2 Pathway | 5.36 | 0.22 | 2.324 | CCR3,HLA-DOA,ICAM1,CD3E,HLA-A,CD4,HLA-DQB1,JUN,HLA-DMB,TIMD4,ICOSLG/LOC102723996,IL2RB,RUNX3,IL2RG,PRKCQ,IKZF1,FGFR2,TLR9,STAT4,CD40,CD80,ICOS,CD86,HLA-DOB,PIK3CD,VAV1,HLA-DRB5 |
| Role of NFAT in Regulation of the Immune Response | 5.3 | 0.2 | 4.017 | BLNK,HLA-DOA,CAMK4,CD3E,HLA-A,NFATC3,NFKBIE,CD4,HLA-DQB1,JUN,MAPK3,HLA-DMB,ITK,PRKCQ,CD79B,FCGR2A,GNG2,GNAI1,FGFR2,CD79A,TLR9,PLCB4,CD80,PLCG2,SYK,LYN,CD86,HLA-DOB,MEF2C,PIK3CD,HLA-DRB5,LCP2 |
| Altered T Cell and B Cell Signaling in Rheumatoid Arthritis | 5.28 | 0.26 | #NUM! | Tlr11,HLA-DOA,SPP1,CD79B,HLA-A,LTB,CD79A,HLA-DQB1,TLR9,TNFRSF13C,CD80,CD40,CXCL13,HLA-DMB,TLR1,CCL21,CD86,HLA-DOB,TNFRSF13B,HLA-DRB5 |
| Role of BRCA1 in DNA Damage Response | 5.16 | 0.264 | 0.535 | FANCM,TP53,ATF1,TOPBP1,SMARCE1,RBL1,FANCL,RAD50,MLH1,CHEK1,CDKN1A,BRCA2,STAT1,BRCA1,BRIP1,BLM,E2F2,E2F8,PHF10 |
| Leukocyte Extravasation Signaling | 5.06 | 0.187 | 2.121 | RAC2,ICAM1,PTK2B,TIMP1,SIPA1,CTNNB1,MMP12,ACTA1,ITGA4,ITK,VCAM1,PRKCQ,CLDN18,ARHGAP4,GNAI1,THY1,FGFR2,MMP2,NCF4,TLR9,ITGAL,RAP1A,ROCK1,ARHGAP5,F11R,WIPF1,WAS,PLCG2,RASGRP1,VAV1,PIK3CD,CTTN,CLDN3,ARHGAP8/PRR5-ARHGAP8,PRKCB |
| OX40 Signaling Pathway | 5.04 | 0.306 | -0.816 | H2-T24,HLA-DOA,CD3E,HLA-A,CD4,NFKBIE,HLA-DQB1,BCL2,HLA-G,JUN,HLA-DMB,HLA-DOB,TRAF5,HLA-DRB5,H2-Q8 |
| Crosstalk between Dendritic Cells and Natural Killer Cells | 5.03 | 0.269 | #NUM! | IL2RG,TYROBP,KLRD1,HLA-A,LTB,CD83,TLR9,ITGAL,HLA-G,CSF2RB,CD80,CD40,CD226,CD86,ACTA1,HLA-DRB5,CCR7,IL2RB |
| T Helper Cell Differentiation | 4.92 | 0.274 | #NUM! | HLA-DOA,IL2RG,HLA-A,IL21R,HLA-DQB1,STAT4,CD80,CD40,NGFR,HLA-DMB,ICOS,IL10RA,CD86,HLA-DOB,STAT1,HLA-DRB5,ICOSLG/LOC102723996 |
| Estrogen-mediated S-phase Entry | 4.68 | 0.4 | 1.667 | MYC,CCNA2,CCNE1,TFDP1,CDKN1A,RBL1,CCND1,E2F2,E2F8,SKP2 |
| Heme Biosynthesis II | 4.57 | 0.667 | #NUM! | UROD,UROS,FECH,ALAS2,CPOX,HMBS |
| Allograft Rejection Signaling | 4.56 | 0.333 | #NUM! | HLA-G,HLA-DOA,H2-T24,CD40,CD80,HLA-A,HLA-DMB,CD86,HLA-DOB,HLA-DQB1,HLA-DRB5,H2-Q8 |
| Antigen Presentation Pathway | 4.51 | 0.385 | #NUM! | HLA-G,HLA-DOA,HLA-A,HLA-DMB,CIITA,HLA-DOB,HLA-DQB1,CD74,TAP1,HLA-DRB5 |
| Dendritic Cell Maturation | 4.41 | 0.19 | 3.402 | HLA-DOA,ICAM1,HLA-A,LEPR,NFKBIE,LTB,CD83,HLA-DQB1,NGFR,MAPK3,HLA-DMB,STAT1,TYROBP,FCGR2A,FGFR2,PLCL2,TLR9,STAT4,PLCB4,CD40,CD80,PLCG2,CD86,HLA-DOB,PIK3CD,STAT2,IRF8,HLA-DRB5,CCR7 |
| GADD45 Signaling | 4.24 | 0.444 | #NUM! | TP53,PCNA,CCNE1,CCND3,CDKN1A,BRCA1,CCND1,CCNB1 |
| Autoimmune Thyroid Disease Signaling | 4.18 | 0.357 | #NUM! | HLA-G,HLA-DOA,CD40,CD80,HLA-A,HLA-DMB,CD86,HLA-DOB,HLA-DQB1,HLA-DRB5 |
| PKCθ Signaling in T Lymphocytes | 4.11 | 0.204 | 3.411 | RAC2,HLA-DOA,PRKCQ,CD3E,HLA-A,NFATC3,CD4,NFKBIE,FGFR2,HLA-DQB1,TLR9,JUN,CD80,GRAP2,MAPK3,PLCG2,HLA-DMB,CD86,HLA-DOB,PIK3CD,VAV1,HLA-DRB5,LCP2 |
| PI3K Signaling in B Lymphocytes | 4.02 | 0.197 | 3.962 | BLNK,CD19,CAMK4,ATF1,CD79B,NFATC3,NFKBIE,CD79A,PLCL2,PTPRC,BLK,PLCB4,JUN,CD180,CD40,DAPP1,MAPK3,SYK,PLCG2,LYN,PIK3CD,VAV1,PRKCB,CR2 |
| IL-8 Signaling | 3.9 | 0.173 | 1.826 | RAC2,ICAM1,PTK2B,CCND1,BCL2,HMOX1,JUN,CCND3,MAPK3,MYL12B,CR2,EGFR,VCAM1,NOX4,PLD3,PRKCQ,ANGPT1,GNG2,GNAI1,FGFR2,MMP2,TLR9,CSTB,PLD4,ROCK1,CDH1,MPO,PIK3CD,FNBP1,PRKCB,ITGAX |
| Natural Killer Cell Signaling | 3.73 | 0.206 | #NUM! | RAC2,PAK4,PTPN6,PRKCQ,LAIR1,TYROBP,FCGR2A,KLRD1,FGFR2,TLR9,NCR1,MAPK3,SYK,PLCG2,Klra7 (includes others),PIK3CD,VAV1,LCP2,PRKCB,KLRC1 |
| IL-7 Signaling Pathway | 3.7 | 0.224 | 2.183 | IL2RG,FGFR2,TLR9,CCND1,BCL2,MET,PAX5,MYC,IL7R,EBF1,JUN,CCND3,MAPK3,HGF,LYN,PIK3CD,STAT1 |
| Phagosome Maturation | 3.68 | 0.188 | #NUM! | ATP6V1C2,CTSK,NOX4,ATP6V0B,YKT6,PRDX5,HLA-A,PRDX1,TCIRG1,ATP6V0D2,TAP1,CTSZ,CTSV,TUBA1A,MPO,CTSH,CTSS,LAMP1,CTSE,ATP6V1G1,CTSC,ATP6V0E1,HLA-DRB5,EEA1 |
| Phospholipase C Signaling | 3.63 | 0.162 | 3.528 | PEBP1,BLNK,CAMK4,CD3E,NFATC3,TGM2,HMOX1,AHNAK,MAPK3,PLA2G5,MYL12B,ITGA4,ITK,PLD3,PRKCQ,HDAC4,CD79B,FCGR2A,GNG2,PLA2G1B,CD79A,RAP1A,PLD4,PLCB4,PLA2G2D,GRAP2,PLCG2,SYK,ARHGEF6,LYN,MEF2C,LCP2,FNBP1,PRKCB |
| Nur77 Signaling in T Lymphocytes | 3.52 | 0.267 | #NUM! | HLA-DOA,CAMK4,CASP9,CD80,CD3E,HLA-A,HLA-DMB,CD86,HLA-DOB,HLA-DQB1,HLA-DRB5,BCL2 |
| TREM1 Signaling | 3.49 | 0.231 | 1.807 | Tlr11,ICAM1,TYROBP,CIITA,LAT2,CD83,TLR9,MPO,CD40,MAPK3,PLCG2,TLR1,CASP1,CD86,ITGAX |
| Phagosome Formation | 3.46 | 0.193 | #NUM! | MRC1,Tlr11,FN1,PRKCQ,FCGR2A,FGFR2,PLCL2,TLR9,CLEC7A,PLCB4,PLCG2,SYK,TLR1,PIK3CD,FNBP1,ITGA4,PRKCB,CR2,ITGAX,FCAMR,FCER2 |
| Aryl Hydrocarbon Receptor Signaling | 3.43 | 0.184 | 0.728 | TP53,GSTM1,NFIC,TFDP1,GSTM5,POLA1,GSTM3,RBL1,CCND1,CYP1B1,CHEK1,TGM2,MYC,CCNA2,CCNE1,JUN,CCND3,MAPK3,CDKN1A,HSP90AA1,ALDH18A1,DHFR,HSPB1 |
| Hereditary Breast Cancer Signaling | 3.38 | 0.183 | #NUM! | FANCM,TP53,NPM1,HDAC4,SMARCE1,Ubb,FGFR2,POLR2B,TLR9,CCND1,FANCL,RAD50,MLH1,CHEK1,CCNB1,H2AFX,CDKN1A,BRCA2,PIK3CD,UBC,BRCA1,BLM,PHF10 |
| Agranulocyte Adhesion and Diapedesis | 3.33 | 0.172 | #NUM! | SELL,ICAM1,FN1,MYH8,CCL17,Cxcl9,CXCL10,ICAM2,CXCL13,MMP12,Ccl6,ACTA1,MYH1,ITGA4,VCAM1,Cxcl15,Ppbp,MYH14,CLDN18,GNAI1,MMP2,C5,Ccl9,Glycam1,CCL21,CLDN3 |
| Granulocyte Adhesion and Diapedesis | 3.32 | 0.175 | #NUM! | SELL,ICAM1,CCL17,Cxcl9,CXCL10,ICAM2,CXCL13,NGFR,MMP12,Ccl6,ITGA4,VCAM1,SDC1,Cxcl15,Ppbp,CLDN18,GNAI1,THY1,MMP2,ITGAL,C5,Ccl9,CCL21,CLDN3,HSPB1 |
| p53 Signaling | 3.28 | 0.196 | -0.243 | TP53,PRKDC,TOPBP1,PLAGL1,PERP,FGFR2,TLR9,CCND1,CHEK1,BCL2,SERPINE2,PCNA,JUN,CDKN1A,PIK3CD,BRCA1,PML,CTNNB1,DRAM1 |
| T Cell Receptor Signaling | 3.28 | 0.196 | #NUM! | PTPN7,PRKCQ,CAMK4,CD3E,NFATC3,CD4,CSK,FGFR2,TLR9,CD8B,PTPRC,JUN,GRAP2,MAPK3,RASGRP1,PIK3CD,VAV1,LCP2,ITK |
| Cdc42 Signaling | 3.24 | 0.186 | 0.302 | PAK4,FGD3,H2-T24,HLA-DOA,CD3E,HLA-A,HLA-DQB1,HLA-G,WIPF1,JUN,ACTR3,WAS,HLA-DMB,ARHGEF6,HLA-DOB,VAV1,HLA-DRB5,MYL12B,ITGA4,ITK,H2-Q8 |
| DNA Double-Strand Break Repair by Homologous Recombination | 3.19 | 0.429 | #NUM! | LIG1,GEN1,POLA1,BRCA2,BRCA1,RAD50 |
| Pyrimidine Deoxyribonucleotides De Novo Biosynthesis I | 3.01 | 0.35 | #NUM! | TYMS,CMPK2,RRM2,CMPK1,PCK1,NME7,RRM1 |
| MIF Regulation of Innate Immunity | 2.97 | 0.263 | 0.632 | TP53,LY96,JUN,PLA2G2D,MAPK3,NFKBIE,PLA2G5,PLA2G1B,CD14,CD74 |
| Thyroid Cancer Signaling | 2.97 | 0.263 | #NUM! | TP53,MYC,CDH1,KLK3,MAPK3,LEF1,CTNNB1,TCF3,CCND1,Tcf7 |
| Graft-versus-Host Disease Signaling | 2.95 | 0.281 | #NUM! | HLA-G,HLA-DOA,CD80,HLA-A,HLA-DMB,CD86,HLA-DOB,HLA-DQB1,HLA-DRB5 |
| Fcγ Receptor-mediated Phagocytosis in Macrophages and Monocytes | 2.93 | 0.193 | 2.183 | RAC2,PRKCQ,PLD3,PTK2B,FCGR2A,FYB1,PLD4,HMOX1,ACTR3,WAS,MAPK3,SYK,LYN,VAV1,ACTA1,LCP2,PRKCB |
| Systemic Lupus Erythematosus Signaling | 2.84 | 0.162 | #NUM! | CAMK4,CD3E,NFATC3,HLA-A,HLA-G,PTPRC,JUN,MAPK3,CD22,PRPF40A,PTPN6,CD79B,FCGR2A,FGFR2,CD79A,TLR9,TNFRSF13C,C5,CD80,CD40,PLCG2,LYN,SNRNP70,CD86,PIK3CD |
| Neuroinflammation Signaling Pathway | 2.83 | 0.142 | 2.466 | HLA-DOA,ICAM1,KLK3,AGER,HLA-A,NFATC3,HLA-DQB1,BCL2,CXCL10,HMOX1,JUN,MAPK3,PLA2G5,TLR1,HLA-DMB,CASP1,CASP8,CTNNB1,STAT1,BIRC3,Tlr11,NOX4,VCAM1,TYROBP,PLA2G1B,FGFR2,TLR9,CSF1R,CD40,PLA2G2D,CD80,PLCG2,SYK,HLA-DOB,CD86,PIK3CD,S100G,HLA-DRB5 |
| HER-2 Signaling in Breast Cancer | 2.68 | 0.195 | #NUM! | TP53,PRKCQ,FGFR2,ERBB3,MMP2,TLR9,CCND1,AREG,CCNE1,CASP9,CDKN1A,TSC2,PIK3CD,PRKCB,EGFR |
| Agrin Interactions at Neuromuscular Junction | 2.63 | 0.206 | -0.577 | RAC2,PAK4,JUN,MAPK3,ARHGEF6,ERBB3,DAG1,AGRN,CTTN,ITGAL,ACTA1,ITGA4,EGFR |
| Heme Biosynthesis from Uroporphyrinogen-III I | 2.63 | 0.75 | #NUM! | UROD,FECH,CPOX |
| Glutathione-mediated Detoxification | 2.61 | 0.304 | #NUM! | GSTM1,GSTM5,GSTM3,HPGDS,Gsta4,Gstt1,ANPEP |
| Acute Myeloid Leukemia Signaling | 2.61 | 0.186 | 1.604 | RUNX1,FGFR2,TCF3,TLR9,CCND1,CSF1R,KITLG,MYC,CSF2RB,PIM1,MAPK3,CEBPA,LEF1,PIK3CD,PML,Tcf7 |
| Pancreatic Adenocarcinoma Signaling | 2.6 | 0.173 | 0.535 | TP53,PLD3,TFDP1,FGFR2,TLR9,CCND1,BCL2,PLD4,HMOX1,CCNE1,CASP9,MAPK3,CDKN1A,BRCA2,PIK3CD,STAT1,E2F8,E2F2,EGFR |
| Hepatic Fibrosis / Hepatic Stellate Cell Activation | 2.56 | 0.153 | #NUM! | ICAM1,FN1,LEPR,MYH8,BCL2,COL6A1,TIMP1,HGF,NGFR,LBP,STAT1,EGFR,MYH1,VCAM1,COL4A1,COL6A2,MYH14,FGFR2,MMP2,MET,LY96,CD40,IL10RA,CCL21,CD14,CCR7 |
| MIF-mediated Glucocorticoid Regulation | 2.51 | 0.267 | 1.414 | LY96,PLA2G2D,MAPK3,NFKBIE,PLA2G5,PLA2G1B,CD14,CD74 |
| Endometrial Cancer Signaling | 2.48 | 0.207 | #NUM! | TP53,MYC,CDH1,CASP9,MAPK3,FGFR2,LEF1,PIK3CD,TLR9,CTNNB1,CCND1,MLH1 |
| Prostate Cancer Signaling | 2.45 | 0.18 | #NUM! | TP53,TFDP1,KLK3,NFKBIE,FGFR2,TLR9,CCND1,BCL2,CCNE1,CASP9,MAPK3,CDKN1A,HSP90AA1,LEF1,PIK3CD,CTNNB1 |
| UVA-Induced MAPK Signaling | 2.4 | 0.178 | 2.84 | TP53,ZC3HAV1,FGFR2,PARP8,PLCL2,TLR9,PARP9,PARP1,PLCB4,JUN,CASP9,MAPK3,PLCG2,PIK3CD,STAT1,EGFR |
| Calcium-induced T Lymphocyte Apoptosis | 2.39 | 0.212 | 3.317 | HLA-DOA,CAMK4,PRKCQ,CD3E,HLA-A,CD4,HLA-DMB,HLA-DOB,HLA-DQB1,HLA-DRB5,PRKCB |
| IL-4 Signaling | 2.36 | 0.187 | #NUM! | PTPN6,HLA-DOA,IL2RG,IRF4,NFATC3,HLA-A,FGFR2,HLA-DQB1,TLR9,HLA-DMB,HLA-DOB,PIK3CD,HLA-DRB5,FCER2 |
| Complement System | 2.33 | 0.25 | -0.816 | C4A/C4B,CFD,CD55,C1QC,C1QB,C5,ITGAX,CR2 |
| Sperm Motility | 2.32 | 0.167 | 3.5 | PRKACB,SLC16A10,PDE2A,PRKCQ,CAMK4,PTK2B,GUCY1A3,PLA2G1B,PLCL2,CNGA1,PLBD1,PLCB4,PRKG1,PLA2G2D,NPR1,PLCG2,PLA2G5,PRKCB |
| Cell Cycle: G1/S Checkpoint Regulation | 2.29 | 0.197 | -0.632 | TP53,MYC,CCNE1,HDAC4,TFDP1,CCND3,CDKN1A,RBL1,CCND1,E2F2,E2F8,SKP2 |
| Glucocorticoid Receptor Signaling | 2.28 | 0.132 | #NUM! | PRKACB,ICAM1,CD3E,HSPA14,POU2F2,SGK1,NFATC3,KRT7,NFKBIE,PBX1,SLPI,GTF2H2,POLR2B,BCL2,JUN,KRT13,ANXA1,MAPK3,CEBPA,TAF3,STAT1,KRT4,VCAM1,SMARCE1,FGFR2,KRT80,PCK1,TLR9,SCGB1A1,KRT8,TAF5,DUSP1,KRT19,CDKN1A,HSP90AA1,KRT18,PIK3CD,PHF10 |
| Tetrapyrrole Biosynthesis II | 2.26 | 0.6 | #NUM! | UROS,ALAS2,HMBS |
| Caveolar-mediated Endocytosis Signaling | 2.23 | 0.194 | #NUM! | ALB,CD55,ITSN1,HLA-A,ITGA8,CD48,CAVIN1,ITGAL,ACTA1,ITGA4,ITGAX,EGFR |
| GP6 Signaling Pathway | 2.22 | 0.16 | 0.471 | LAMA5,COL4A1,PRKCQ,CAMK4,APBB1IP,COL6A2,FGFR2,TLR9,LAMC2,LAMB2,COL6A1,GRAP2,LAMB3,PLCG2,SYK,LYN,PIK3CD,LCP2,PRKCB |
| p70S6K Signaling | 2.18 | 0.158 | 2.982 | CD19,F2RL2,IL2RG,PRKCQ,CD79B,GNAI1,FGFR2,CD79A,PLCL2,TLR9,PLCB4,MAPK3,PLCG2,SYK,LYN,PIK3CD,EEF2K,PRKCB,EGFR |
| Type I Diabetes Mellitus Signaling | 2.17 | 0.168 | 1.134 | HLA-DOA,CD3E,HLA-A,NFKBIE,HLA-DQB1,BCL2,HLA-G,CASP9,CD80,NGFR,HLA-DMB,CD86,HLA-DOB,CASP8,STAT1,HLA-DRB5 |
| Molecular Mechanisms of Cancer | 2.16 | 0.125 | #NUM! | PRKACB,RAC2,BMP3,NFKBIE,RBL1,CCND1,CHEK1,BCL2,MYC,CDK8,CASP9,JUN,CCND3,MAPK3,CDK17,CASP8,CTNNB1,BRCA1,BIRC3,E2F8,E2F2,ITGA4,BMP1,TP53,PRKDC,PAK4,PRKCQ,TFDP1,SMAD6,GNAI1,FGFR2,TCF3,TLR9,RAP1A,PLCB4,CDH1,CCNE1,RASGRP1,ARHGEF6,CDKN1A,LEF1,PIK3CD,FNBP1,LRP1,PRKCB |
| Retinol Biosynthesis | 2.16 | 0.235 | #NUM! | CEL,PNLIPRP2,LPL,PNLIPRP1,RDH12,RBP1,PNPLA2,PNLIP |
| Aldosterone Signaling in Epithelial Cells | 2.1 | 0.149 | 0.535 | CRYAB,PRKCQ,DNAJC9,HSPA14,SGK1,HSPH1,FGFR2,PLCL2,TLR9,SCNN1A,PLCB4,SCNN1G,DUSP1,HSCB,MAPK3,PLCG2,HSP90AA1,PIK3CD,SCNN1B,PIP4K2A,PRKCB,HSPB1 |
| Role of Pattern Recognition Receptors in Recognition of Bacteria and Viruses | 2.1 | 0.156 | 1.604 | Tlr11,PRKCQ,OAS2,FGFR2,C1QC,C1QB,OAS3,EIF2S1,TLR9,RNASEL,C5,CLEC7A,MAPK3,PLCG2,SYK,TLR1,CASP1,PIK3CD,PRKCB |
| Role of JAK1 and JAK3 in γc Cytokine Signaling | 2.06 | 0.185 | #NUM! | BLNK,IL7R,IL2RG,PTK2B,MAPK3,SYK,IL21R,FGFR2,PIK3CD,TLR9,STAT1,IL2RB |
| Cyclins and Cell Cycle Regulation | 2.06 | 0.178 | 2.111 | TP53,HDAC4,TFDP1,CCNB2,CCND1,CCNB1,SKP2,CCNA2,CCNE1,CCND3,CDKN1A,E2F8,E2F2 |
| Tumoricidal Function of Hepatic Natural Killer Cells | 2.05 | 0.273 | #NUM! | SERPINB9,ICAM1,CASP9,LYVE1,CASP8,ITGAL |
| LXR/RXR Activation | 2.02 | 0.159 | -1.508 | C4A/C4B,FDFT1,ALB,LYZ,LY96,TF,ITIH4,SREBF1,NGFR,FASN,LPL,CD14,ACACA,PLTP,LBP,HADH,RBP4 |
| Glioma Signaling | 2.02 | 0.159 | 1 | TP53,PRKCQ,CAMK4,TFDP1,FGFR2,RBL1,TLR9,IGF2R,CCND1,MAPK3,PLCG2,CDKN1A,PIK3CD,E2F8,E2F2,PRKCB,EGFR |
| UVB-Induced MAPK Signaling | 1.96 | 0.186 | 1 | TP53,H3F3A/H3F3B,PRKCQ,JUN,MAPK3,HIST1H3C,FGFR2,PIK3CD,TLR9,PRKCB,EGFR |
| BER pathway | 1.96 | 0.364 | #NUM! | LIG1,PCNA,POLE,PARP1 |
| GM-CSF Signaling | 1.96 | 0.179 | 0.577 | RUNX1,CSF2RB,PIM1,MAPK3,LYN,FGFR2,PIK3CD,TLR9,BCL2A1,STAT1,CCND1,PRKCB |
| Acute Phase Response Signaling | 1.88 | 0.142 | -0.832 | FN1,Saa3,NFKBIE,VWF,CP,TCF3,RBP1,C5,C4A/C4B,HMOX1,ALB,HP,FTL,JUN,F8,TF,ITIH4,MAPK3,NGFR,PIK3CD,LBP,RBP4 |
| ATM Signaling | 1.84 | 0.163 | 0.577 | TP53,SMC3,ATF1,TOPBP1,CCNB2,RAD50,CHEK1,CCNB1,JUN,SMC2,H2AFX,CDKN1A,BRCA1,BLM |
| DNA damage-induced 14-3-3σ Signaling | 1.81 | 0.278 | #NUM! | TP53,CCNE1,CCNB2,BRCA1,CCNB1 |
| Phospholipases | 1.81 | 0.185 | #NUM! | PLBD1,PLD4,HMOX1,PLCB4,PLD3,PLA2G2D,PLCG2,PLA2G5,PLA2G1B,PLCL2 |
| Endothelin-1 Signaling | 1.79 | 0.138 | 2.711 | PRKCQ,PLD3,GUCY1A3,GNAI1,PLA2G1B,FGFR2,PLCL2,TLR9,PLBD1,MYC,PLD4,HMOX1,PLCB4,JUN,CASP9,PLA2G2D,MAPK3,PLCG2,PLA2G5,CASP1,PIK3CD,CASP8,PRKCB |
| Role of PKR in Interferon Induction and Antiviral Response | 1.79 | 0.205 | #NUM! | TP53,CASP9,NFKBIE,TRAF5,CASP8,STAT1,EIF2S1,RNASEL |
| CTLA4 Signaling in Cytotoxic T Lymphocytes | 1.78 | 0.165 | #NUM! | PTPN6,CD3E,HLA-A,FGFR2,TLR9,CD8B,CD80,GRAP2,SYK,CD86,PIK3CD,PTPN22,LCP2 |
| Small Cell Lung Cancer Signaling | 1.78 | 0.165 | #NUM! | MYC,TP53,CCNE1,CASP9,TFDP1,NFKBIE,FGFR2,PIK3CD,TRAF5,TLR9,CCND1,BCL2,SKP2 |
| Antioxidant Action of Vitamin C | 1.78 | 0.156 | -1.941 | PLD3,NFKBIE,PLA2G1B,PLCL2,GLRX,PLBD1,PLD4,CSF2RB,HMOX1,PLCB4,PLA2G2D,MAPK3,PLCG2,PLA2G5,TXNRD2 |
| Cell Cycle: G2/M DNA Damage Checkpoint Regulation | 1.77 | 0.191 | -0.707 | TP53,PRKDC,CDKN1A,TOP2A,CCNB2,BRCA1,CHEK1,CCNB1,SKP2 |
| Virus Entry via Endocytic Pathways | 1.76 | 0.159 | #NUM! | RAC2,PRKCQ,ITSN1,HLA-A,FGFR2,TLR9,ITGAL,CD55,PLCG2,TFRC,PIK3CD,ACTA1,PRKCB,ITGA4 |
| Tec Kinase Signaling | 1.75 | 0.14 | 2.982 | PAK4,PRKCQ,PTK2B,GNG2,GNAI1,FGFR2,TLR9,STAT4,BLK,WAS,PLCG2,LYN,STAT2,VAV1,PIK3CD,STAT1,FNBP1,ACTA1,ITGA4,PRKCB,ITK |
| FcγRIIB Signaling in B Lymphocytes | 1.71 | 0.188 | 2.333 | BLNK,CD79B,PLCG2,SYK,LYN,FGFR2,CD79A,PIK3CD,TLR9 |
| Integrin Signaling | 1.7 | 0.132 | 1.279 | RAC2,PAK4,ITGA8,FGFR2,TSPAN2,GSN,TLR9,ITGAL,RAP1A,ARHGAP5,ROCK1,PARVB,WIPF1,ACTR3,WAS,PLCG2,MAPK3,PIK3CD,CTTN,NEDD9,ACTA1,MYL12B,FNBP1,ITGA4,ITGAX |
| Fc Epsilon RI Signaling | 1.69 | 0.15 | #NUM! | RAC2,PRKCQ,PLA2G1B,FGFR2,TLR9,PLA2G2D,GRAP2,MAPK3,PLCG2,SYK,PLA2G5,LYN,PIK3CD,VAV1,LCP2,PRKCB |
| DNA Double-Strand Break Repair by Non-Homologous End Joining | 1.68 | 0.308 | #NUM! | PRKDC,WRN,RAD50,PARP1 |
| nNOS Signaling in Skeletal Muscle Cells | 1.68 | 0.308 | #NUM! | CAMK4,SNTB1,DMD,DAG1 |
| ERK/MAPK Signaling | 1.63 | 0.133 | 2.294 | PRKACB,PPP1R14C,RAC2,PAK4,PTK2B,ATF1,HIST1H3C,PLA2G1B,FGFR2,TLR9,RAP1A,MYC,H3F3A/H3F3B,PLA2G2D,DUSP1,MAPK3,PLCG2,PLA2G5,PIK3CD,STAT1,ITGA4,PRKCB,HSPB1 |
| LPS/IL-1 Mediated Inhibition of RXR Function | 1.59 | 0.13 | 0.302 | GSTM1,GSTM5,CHST7,ACOX1,GSTM3,PAPSS2,IL4I1,CHST2,LY96,JUN,SMOX,SREBF1,NGFR,CAT,CHST3,HS6ST2,FABP1,CD14,CYP2A6 (includes others),ALDH18A1,PLTP,LBP,NDST1,CYP4A11 |
| Death Receptor Signaling | 1.58 | 0.155 | 1.387 | ROCK1,CASP9,NFKBIE,ZC3HAV1,PARP8,CASP8,BIRC3,PARP9,ACTA1,ARHGDIB,BCL2,PARP1,HSPB1 |
| Purine Nucleotides Degradation II (Aerobic) | 1.57 | 0.286 | #NUM! | NT5C3A,GDA,XDH,IMPDH1 |
| Actin Cytoskeleton Signaling | 1.53 | 0.128 | 0.853 | RAC2,FGD3,FN1,MYH8,CYFIP2,ACTR3,MAPK3,LBP,MYL12B,ACTA1,MYH1,ITGA4,PAK4,MYH14,CSK,FGFR2,TLR9,GSN,ROCK1,WAS,ARHGEF6,CD14,PIK3CD,VAV1,PIP4K2A |
| Role of Macrophages, Fibroblasts and Endothelial Cells in Rheumatoid Arthritis | 1.52 | 0.12 | #NUM! | ICAM1,FN1,CAMK4,NFATC3,NFKBIE,PRSS2,LTB,CCND1,MYC,JUN,NGFR,MAPK3,TLR1,CEBPA,TRAF5,CTNNB1,PRSS3,Prss1 (includes others),Tlr11,VCAM1,PRKCQ,FGFR2,PLCL2,TLR9,TCF3,C5,ROCK1,PLCB4,PLCG2,PIK3CD,LEF1,Tcf7,LRP1,PRKCB |
| CCR5 Signaling in Macrophages | 1.51 | 0.167 | #NUM! | CAMK4,PRKCQ,JUN,PTK2B,CD3E,PLCG2,CD4,GNG2,GNAI1,PRKCB |
| Thrombopoietin Signaling | 1.51 | 0.167 | 1.265 | MYC,PRKCQ,JUN,MAPK3,PLCG2,FGFR2,PIK3CD,TLR9,STAT1,PRKCB |
| Role of PI3K/AKT Signaling in the Pathogenesis of Influenza | 1.51 | 0.167 | 0 | KPNA3,CASP9,MAPK3,NFKBIE,GNAI1,FGFR2,PIK3CD,TLR9,PLAC8,MLH1 |
| CCR3 Signaling in Eosinophils | 1.49 | 0.142 | #NUM! | CCR3,PAK4,PRKCQ,CAMK4,GNG2,GNAI1,PLA2G1B,FGFR2,TLR9,ROCK1,PLCB4,PLA2G2D,MAPK3,PLA2G5,PIK3CD,PRKCB |
| Gαq Signaling | 1.49 | 0.133 | 2.524 | RGS18,PRKCQ,PLD3,CAMK4,PTK2B,NFATC3,CSK,NFKBIE,GNG2,FGFR2,TLR9,ROCK1,PLD4,HMOX1,PLCB4,MAPK3,PLCG2,PIK3CD,FNBP1,PRKCB |
| Regulation of Actin-based Motility by Rho | 1.47 | 0.154 | 1.508 | ROCK1,RAC2,PAK4,WIPF1,ACTR3,WAS,GSN,PIP4K2A,FNBP1,ACTA1,MYL12B,ITGA4 |
| Glioblastoma Multiforme Signaling | 1.47 | 0.132 | 1.213 | TP53,FGFR2,PLCL2,TCF3,TLR9,CCND1,MYC,CCNE1,PLCB4,MAPK3,PLCG2,CDKN1A,TSC2,LEF1,PIK3CD,CTNNB1,FNBP1,E2F2,E2F8,EGFR |
| Granzyme B Signaling | 1.46 | 0.267 | 2 | PRKDC,CASP9,CASP8,PARP1 |
| Production of Nitric Oxide and Reactive Oxygen Species in Macrophages | 1.46 | 0.129 | 1.147 | PPP1R14C,PTPN6,PRKCQ,NFKBIE,FGFR2,NCF4,TLR9,RAP1A,ALB,LYZ,JUN,MPO,MAPK3,NGFR,PLCG2,CAT,PIK3CD,IRF8,STAT1,FNBP1,PRKCB,RBP4 |
| Renal Cell Carcinoma Signaling | 1.45 | 0.157 | -0.333 | MET,PAK4,JUN,MAPK3,HGF,Ubb,FGFR2,PIK3CD,UBC,TLR9,RAP1A |
| Tryptophan Degradation III (Eukaryotic) | 1.45 | 0.227 | #NUM! | KMO,HAAO,CA1,HSD17B4,HADH |
| p38 MAPK Signaling | 1.43 | 0.142 | 1.604 | TP53,TIFA,ATF1,HIST1H3C,PLA2G1B,MYC,H3F3A/H3F3B,PLA2G2D,DUSP1,PLA2G5,MAP4K1,MEF2C,EEF2K,STAT1,HSPB1 |
| Ovarian Cancer Signaling | 1.42 | 0.134 | #NUM! | PRKACB,TP53,TFDP1,FGFR2,MMP2,TCF3,TLR9,CCND1,MLH1,BCL2,MAPK3,BRCA2,PIK3CD,LEF1,BRCA1,CTNNB1,Tcf7,EGFR |
| CD40 Signaling | 1.41 | 0.155 | -0.302 | ICAM1,JUN,CD40,ATF1,MAPK3,NFKBIE,FGFR2,PIK3CD,TRAF5,TLR9,FCER2 |
| Heme Degradation | 1.4 | 0.5 | #NUM! | HMOX1,BLVRB |
| α-tocopherol Degradation | 1.4 | 0.5 | #NUM! | CYP4A11,CYP4F2 |
| Melatonin Degradation II | 1.4 | 0.5 | #NUM! | SMOX,IL4I1 |
| dTMP De Novo Biosynthesis | 1.4 | 0.5 | #NUM! | TYMS,DHFR |
| FAK Signaling | 1.4 | 0.146 | #NUM! | PAK4,HMMR,CSK,FGFR2,TLR9,WAS,MAPK3,PLCG2,ARHGEF6,PIK3CD,ACTA1,EGFR,ITGA4 |
| Cytotoxic T Lymphocyte-mediated Apoptosis of Target Cells | 1.37 | 0.217 | 0 | CASP9,CD3E,HLA-A,CASP8,BCL2 |
| Cellular Effects of Sildenafil (Viagra) | 1.37 | 0.139 | #NUM! | PRKACB,PDE2A,CAMK4,GUCY1A3,MYH8,MYH14,PLCL2,PLCB4,CACNA1E,PRKG1,PABPC4,PLCG2,ACTA1,MYL12B,MYH1 |
| autophagy | 1.32 | 0.161 | #NUM! | CTSZ,CTSV,CTSK,CTSH,CTSS,LAMP1,CTSE,CTSC,BCL2 |
| Gα12/13 Signaling | 1.32 | 0.134 | 0.258 | F2RL2,PTK2B,NFKBIE,FGFR2,TLR9,CDH11,ROCK1,CDH1,JUN,MAPK3,CDH8,VAV1,MEF2C,PIK3CD,CTNNB1,MYL12B |
| Role of CHK Proteins in Cell Cycle Checkpoint Control | 1.3 | 0.167 | 0.378 | TP53,PCNA,CDKN1A,BRCA1,E2F2,E2F8,RAD50,CHEK1 |
| Unfolded protein response | 1.3 | 0.167 | #NUM! | SYVN1,P4HB,HSPA14,SREBF1,HSPH1,ERO1B,CEBPA,BCL2 |
| Macropinocytosis Signaling | 1.3 | 0.149 | 1.667 | MET,MRC1,PRKCQ,PLCG2,HGF,CD14,FGFR2,PIK3CD,TLR9,CSF1R,PRKCB |
| VEGF Signaling | 1.3 | 0.141 | 0.577 | SH2D2A,PTPN6,PTK2B,FGFR2,EIF2S1,TLR9,BCL2,ROCK1,MAPK3,PLCG2,PIK3CD,ACTA1,PRKCB |
|  |  |  |  |  |

**Supplementary Table 4** 283 molecules related with lung tumors were identified between *Kras^LA2^* and *miR-301a^-/-^;Kras^LA2^* mice.

| Entrez Gene Name | Ensembl/Gene Symbol - mouse | Expr Log Ratio | Networks | Location | Type(s) |
| --- | --- | --- | --- | --- | --- |
| ATP binding cassette subfamily B member 10 | Abcb10 | 2.502 |  | Cytoplasm | transporter |
| ATP binding cassette subfamily C member 5 | Abcc5 | -1.741 | 3 | Plasma Membrane | transporter |
| acetylcholinesterase (Cartwright blood group) | Ache | 4.435 | 19 | Plasma Membrane | enzyme |
| acyl-CoA oxidase 1 | Acox1 | -2.681 |  | Cytoplasm | enzyme |
| ADAM metallopeptidase domain 15 | Adam15 | -2.145 | 19 | Plasma Membrane | peptidase |
| ADAM metallopeptidase with thrombospondin type 1 motif 14 | Adamts14 | 1.301 |  | Extracellular Space | peptidase |
| adenosine A3 receptor | Adora3 | 1.816 |  | Plasma Membrane | G-protein coupled receptor |
| advanced glycosylation end-product specific receptor | Ager | -3.844 |  | Plasma Membrane | transmembrane receptor |
| agrin | Agrn | -1.856 | 19 | Plasma Membrane | other |
| AHNAK nucleoprotein | Ahnak | -1.337 | 3 | Nucleus | other |
| aldolase, fructose-bisphosphate A | Aldoa | -1.497 | 12 | Cytoplasm | enzyme |
| angiopoietin 1 | Angpt1 | 1.404 | 2 | Extracellular Space | growth factor |
| angiopoietin like 1 | Angptl1 | 2.163 |  | Plasma Membrane | other |
| alanyl aminopeptidase, membrane | Anpep | -1.648 |  | Plasma Membrane | peptidase |
| annexin A1 | Anxa1 | -1.526 | 9 | Plasma Membrane | enzyme |
| annexin A2 | Anxa2 | -2.344 | 8 | Plasma Membrane | other |
| annexin A3 | Anxa3 | -2.097 | 22 | Cytoplasm | enzyme |
| amyloid beta precursor protein binding family B member 1 interacting protein | Apbb1ip | 1.16 |  | Cytoplasm | other |
| amyloid beta precursor like protein 2 | Aplp2 | -1.435 | 19 | Cytoplasm | other |
| amphiregulin | Areg | -4.59 | 7 | Extracellular Space | growth factor |
| Rho GTPase activating protein 5 | Arhgap5 | 1.42 |  | Cytoplasm | enzyme |
| ATPase family, AAA domain containing 2 | Atad2 | 2.144 | 14 | Nucleus | enzyme |
| ATPase Na+/K+ transporting subunit alpha 1 | Atp1a1 | -2.051 |  | Plasma Membrane | transporter |
| ATPase Na+/K+ transporting subunit alpha 2 | Atp1a2 | 1.016 |  | Plasma Membrane | transporter |
| ATPase Na+/K+ transporting subunit beta 1 | Atp1b1 | -1.353 |  | Plasma Membrane | transporter |
| BCL2, apoptosis regulator | Bcl2 | 1.336 |  | Cytoplasm | transporter |
| B-cell CLL/lymphoma 11A | Bcl11a | 2.756 |  | Nucleus | transcription regulator |
| BLK proto-oncogene, Src family tyrosine kinase | Blk | 2.681 |  | Cytoplasm | kinase |
| B-cell linker | Blnk | 2.328 | 18 | Cytoplasm | other |
| BPI fold containing family A member 1 | Bpifa1 | -1.3 |  | Extracellular Space | other |
| BRCA1, DNA repair associated | Brca1 | 2.993 | 2 | Nucleus | transcription regulator |
| BRCA2, DNA repair associated | Brca2 | 2.386 |  | Nucleus | transcription regulator |
| BRCA1 interacting protein C-terminal helicase 1 | Brip1 | 2.377 |  | Nucleus | enzyme |
| BUB1 mitotic checkpoint serine/threonine kinase | Bub1 | 2.413 |  | Nucleus | kinase |
| cathelicidin antimicrobial peptide | Camp | 1.814 |  | Cytoplasm | other |
| caspase recruitment domain family member 6 | Card6 | 1.074 | 21 | Cytoplasm | other |
| caspase 8 | Casp8 | 1.26 | 16 | Nucleus | peptidase |
| catalase | Cat | 1.636 | 15 | Cytoplasm | enzyme |
| coiled-coil domain containing 88A | Ccdc88a | 1.227 |  | Cytoplasm | other |
| C-C motif chemokine ligand 17 | Ccl17 | -3.516 |  | Extracellular Space | cytokine |
| C-C motif chemokine ligand 21 | Gm13304 | -9.434 |  | Extracellular Space | cytokine |
| cyclin A2 | Ccna2 | 3.799 | 7 | Nucleus | other |
| cyclin B1 | Ccnb1 | 3.256 | 7 | Cytoplasm | kinase |
| cyclin B2 | Ccnb2 | 3.324 | 7 | Cytoplasm | kinase |
| cyclin D1 | Ccnd1 | -1.129 | 1 | Nucleus | transcription regulator |
| cyclin D3 | Ccnd3 | 1.496 | 9 | Nucleus | kinase |
| cyclin E1 | Ccne1 | 1.638 | 9 | Nucleus | transcription regulator |
| C-C motif chemokine receptor 6 | Ccr6 | 1.648 |  | Plasma Membrane | G-protein coupled receptor |
| centriole, cilia and spindle associated protein | Ccsap | 1.157 | 2 | Cytoplasm | other |
| CD4 molecule | Cd4 | 2.87 | 9 | Plasma Membrane | transmembrane receptor |
| CD9 molecule | Cd9 | -2.44 |  | Plasma Membrane | other |
| CD48 molecule | Cd48 | 1.139 | 21 | Plasma Membrane | other |
| CD151 molecule (Raph blood group) | Cd151 | -1.575 |  | Plasma Membrane | other |
| CD8a molecule | Cd8a | 2.41 |  | Plasma Membrane | other |
| cell division cycle 27 | Cdc27 | 1.125 | 25 | Nucleus | other |
| cadherin 1 | Cdh1 | -1.297 |  | Plasma Membrane | other |
| cyclin dependent kinase inhibitor 1A | Cdkn1a | -2.351 | 11 | Nucleus | kinase |
| CCAAT/enhancer binding protein alpha | Cebpa | -2.776 |  | Nucleus | transcription regulator |
| centromere protein F | Cenpf | 3.534 | 25 | Nucleus | other |
| centrosomal protein 192 | Cep192 | 1.604 | 12 | Cytoplasm | other |
| CHM, Rab escort protein 1 | Chm | 1.495 |  | Cytoplasm | enzyme |
| class II major histocompatibility complex transactivator | Ciita | 1.121 |  | Nucleus | transcription regulator |
| claudin 3 | Cldn3 | -4.649 | 23 | Plasma Membrane | transmembrane receptor |
| C-type lectin domain containing 7A | Clec7a | -2.309 |  | Plasma Membrane | transmembrane receptor |
| CAP-Gly domain containing linker protein family member 4 | Clip4 | -3.106 |  | Other | other |
| cyclic nucleotide gated channel alpha 1 | Cnga1 | -1.114 | 4 | Plasma Membrane | ion channel |
| cochlin | Coch | 4.624 |  | Extracellular Space | other |
| coproporphyrinogen oxidase | Cpox | 3.42 |  | Cytoplasm | enzyme |
| colony stimulating factor 1 receptor | Csf1r | 1.166 | 18 | Plasma Membrane | kinase |
| CUB and Sushi multiple domains 3 | Csmd3 | 7.079 | 22 | Plasma Membrane | enzyme |
| catenin beta 1 | Ctnnb1 | -3.832 | 10 | Nucleus | transcription regulator |
| catenin delta 2 | Ctnnd2 | 1.425 | 10 | Plasma Membrane | other |
| cathepsin D | Ctsd | -1.715 | 25 | Cytoplasm | peptidase |
| cathepsin Z | Ctsz | -2.172 | 25 | Cytoplasm | peptidase |
| cullin 4B | Cul4b | 1.195 | 2 | Nucleus | other |
| C-X-C motif chemokine ligand 13 | Cxcl13 | 2.347 |  | Extracellular Space | cytokine |
| cytochrome P450 family 1 subfamily B member 1 | Cyp1b1 | 1.989 |  | Cytoplasm | enzyme |
| cytochrome P450 family 2 subfamily A member 6 | Cyp2a5 | -1.155 |  | Cytoplasm | enzyme |
| deoxycytidine kinase | Dck | 2.545 | 25 | Nucleus | kinase |
| dihydrofolate reductase | Dhfr | 1.242 | 7 | Nucleus | enzyme |
| dystrophin | Dmd | 1.221 | 19 | Plasma Membrane | other |
| Dmx like 1 | Dmxl1 | 1.43 |  | Extracellular Space | other |
| DNA methyltransferase 1 | Dnmt1 | 1.139 |  | Nucleus | enzyme |
| dedicator of cytokinesis 8 | Dock8 | 1.081 |  | Cytoplasm | other |
| ELL associated factor 2 | Eaf2 | 2.793 | 2 | Nucleus | transcription regulator |
| EGF like domain multiple 7 | Egfl7 | -1.722 |  | Extracellular Space | other |
| epidermal growth factor receptor | Egfr | 1.45 | 3 | Plasma Membrane | kinase |
| early growth response 3 | Egr3 | 2.505 |  | Nucleus | transcription regulator |
| eukaryotic translation initiation factor 2 subunit alpha | Eif2s1 | 1.336 |  | Cytoplasm | translation regulator |
| elastase, neutrophil expressed | Elane | 6.87 |  | Extracellular Space | peptidase |
| endothelial PAS domain protein 1 | Epas1 | 1.239 |  | Nucleus | transcription regulator |
| epithelial cell adhesion molecule | Epcam | -2.335 | 10 | Plasma Membrane | other |
| EPH receptor B4 | Ephb4 | -1.818 |  | Plasma Membrane | kinase |
| epiplakin 1 | Eppk1 | -3.973 |  | Cytoplasm | other |
| erb-b2 receptor tyrosine kinase 3 | Erbb3 | -1.119 |  | Plasma Membrane | kinase |
| enhancer of zeste 2 polycomb repressive complex 2 subunit | Ezh2 | 1.99 | 15 | Nucleus | transcription regulator |
| family with sequence similarity 129 member C | Fam129c | 1.854 | 2 | Other | other |
| Fanconi anemia complementation group L | Fancl | 1.3 |  | Nucleus | enzyme |
| Fanconi anemia complementation group M | Fancm | 1.588 |  | Nucleus | enzyme |
| fatty acid synthase | Fasn | -2.579 | 20 | Cytoplasm | enzyme |
| FAT atypical cadherin 1 | Fat1 | -1.106 |  | Plasma Membrane | other |
| fructose-bisphosphatase 1 | Fbp1 | -9.285 | 25 | Cytoplasm | phosphatase |
| farnesyl-diphosphate farnesyltransferase 1 | Fdft1 | -1.104 | 20 | Cytoplasm | enzyme |
| fibroblast growth factor receptor 2 | Fgfr2 | -1.113 |  | Plasma Membrane | kinase |
| fibronectin 1 | Fn1 | -2.055 |  | Extracellular Space | enzyme |
| focadhesin | Focad | 1.039 |  | Plasma Membrane | other |
| ferritin light chain | Ftl1 | -1.904 |  | Cytoplasm | enzyme |
| guanylate binding protein 4 | Gbp3 | 1.398 | 5 | Cytoplasm | enzyme |
| glycoprotein nmb | Gpnmb | -4.525 |  | Plasma Membrane | enzyme |
| G protein-coupled receptor 132 | Gpr132 | 1.564 |  | Plasma Membrane | G-protein coupled receptor |
| G protein-coupled receptor 174 | Gpr174 | 2.49 |  | Plasma Membrane | G-protein coupled receptor |
| gelsolin | Gsn | -1.326 |  | Extracellular Space | other |
| glutathione S-transferase mu 1 | Gstm2 | -2.601 |  | Cytoplasm | enzyme |
| guanylate cyclase 1 soluble subunit alpha 2 | Gucy1a2 | 1.346 | 23 | Cytoplasm | enzyme |
| HECT domain and ankyrin repeat containing E3 ubiquitin protein ligase 1 | Hace1 | 1.509 | 6 | Cytoplasm | enzyme |
| hepatocyte growth factor | Hgf | 1.542 |  | Extracellular Space | growth factor |
| histone cluster 1 H3 family member c | Hist2h3c1 | 1.607 |  | Nucleus | other |
| histone cluster 2 H2A family member c | Hist2h2ac | 1.961 | 3 | Nucleus | other |
| major histocompatibility complex, class I, A | H2-Q4 | 1.232 |  | Plasma Membrane | other |
| major histocompatibility complex, class II, DQ beta 1 | H2-Ab1 | 1.15 |  | Plasma Membrane | other |
| major histocompatibility complex, class I, G | H2-M3 | 1.277 |  | Plasma Membrane | other |
| heme oxygenase 1 | Hmox1 | 1.576 |  | Cytoplasm | enzyme |
| heparan sulfate 6-O-sulfotransferase 2 | Hs6st2 | 2.322 | 13 | Plasma Membrane | enzyme |
| hydroxysteroid 17-beta dehydrogenase 4 | Hsd17b4 | -1.038 |  | Cytoplasm | enzyme |
| heat shock protein 90 alpha family class A member 1 | Hsp90aa1 | 1.011 | 16 | Cytoplasm | enzyme |
| intercellular adhesion molecule 1 | Icam1 | -1.449 |  | Plasma Membrane | transmembrane receptor |
| intercellular adhesion molecule 2 | Icam2 | -1.096 |  | Plasma Membrane | other |
| insulin like growth factor 2 receptor | Igf2r | -1.512 |  | Plasma Membrane | transmembrane receptor |
| insulin like growth factor binding protein 2 | Igfbp2 | -3.353 |  | Extracellular Space | other |
| IKAROS family zinc finger 1 | Ikzf1 | 2.009 |  | Nucleus | transcription regulator |
| interleukin 7 receptor | Il7r | 1.576 |  | Plasma Membrane | transmembrane receptor |
| integrin subunit alpha 1 | Itga1 | 1.109 |  | Plasma Membrane | other |
| integrin subunit alpha 4 | Itga4 | 2.204 | 9 | Plasma Membrane | transmembrane receptor |
| integrin subunit alpha L | Itgal | 1.042 |  | Plasma Membrane | transmembrane receptor |
| integrin subunit alpha X | Itgax | -2.354 |  | Plasma Membrane | transmembrane receptor |
| IL2 inducible T-cell kinase | Itk | 1.925 |  | Cytoplasm | kinase |
| Jun proto-oncogene, AP-1 transcription factor subunit | Jun | -1.012 |  | Nucleus | transcription regulator |
| potassium voltage-gated channel subfamily J member 16 | Kcnj16 | 4.081 | 23 | Plasma Membrane | ion channel |
| lysine demethylase 5A | Kdm5a | 1.155 |  | Nucleus | transcription regulator |
| Kell blood group, metallo-endopeptidase | Kel | 6.139 | 17 | Plasma Membrane | peptidase |
| kinesin family member 11 | Kif11 | 3.49 | 6 | Nucleus | other |
| kinesin family member 14 | Kif14 | 2.448 | 14 | Cytoplasm | enzyme |
| kinesin family member 23 | Kif23 | 2.301 | 8 | Cytoplasm | other |
| kinesin family member 21B | Kif21b | 1.205 |  | Cytoplasm | other |
| keratin 7 | Krt7 | -2.919 | 4 | Cytoplasm | other |
| keratin 8 | Krt8 | -3.132 | 4 | Cytoplasm | other |
| keratin 19 | Krt19 | -2.907 | 4 | Cytoplasm | other |
| laminin subunit alpha 5 | Lama5 | -2.916 |  | Extracellular Space | other |
| laminin subunit beta 3 | Lamb3 | -3.722 | 2 | Extracellular Space | transporter |
| lysosomal associated membrane protein 1 | Lamp1 | -1.483 |  | Plasma Membrane | other |
| lipocalin 2 | Lcn2 | -4.506 |  | Extracellular Space | transporter |
| leptin receptor | Lepr | 1.18 | 6 | Plasma Membrane | transmembrane receptor |
| galectin 1 | Lgals1 | -2.352 |  | Extracellular Space | other |
| DNA ligase 1 | Lig1 | 2.563 | 11 | Nucleus | enzyme |
| LDL receptor related protein 1 | Lrp1 | -1.384 | 9 | Plasma Membrane | transmembrane receptor |
| LDL receptor related protein 2 | Lrp2 | -1.941 | 19 | Plasma Membrane | transporter |
| leucine rich repeats and IQ motif containing 1 | Lrriq1 | 1.141 |  | Other | other |
| LYN proto-oncogene, Src family tyrosine kinase | Lyn | 1.035 | 16 | Cytoplasm | kinase |
| mitotic arrest deficient 2 like 1 | Mad2l1 | 2.322 | 7 | Nucleus | other |
| MAF bZIP transcription factor B | Mafb | 2.705 | 15 | Nucleus | transcription regulator |
| MAGE family member D1 | Maged1 | -2.113 | 24 | Plasma Membrane | transcription regulator |
| mitogen-activated protein kinase binding protein 1 | Mapkbp1 | -2.312 |  | Cytoplasm | other |
| myoglobin | Mb | -1.843 |  | Cytoplasm | transporter |
| minichromosome maintenance complex component 2 | Mcm2 | 2.709 |  | Nucleus | enzyme |
| minichromosome maintenance complex component 5 | Mcm5 | 2.222 | 1 | Nucleus | enzyme |
| maternally expressed 3 | Meg3 | -4.532 | 15 | Other | other |
| MET proto-oncogene, receptor tyrosine kinase | Met | -1.016 |  | Plasma Membrane | kinase |
| MINDY lysine 48 deubiquitinase 3 | Fam188a | 1.462 | 1 | Nucleus | enzyme |
| microRNA 142 | Mir142 | 1.483 | 19 | Cytoplasm | microRNA |
| microRNA 486-1 | Mir486 | 1.095 | 22 | Cytoplasm | microRNA |
| marker of proliferation Ki-67 | Mki67 | 4.435 |  | Nucleus | other |
| mutL homolog 1 | Mlh1 | 1.577 |  | Nucleus | enzyme |
| matrix metallopeptidase 2 | Mmp2 | -1.033 | 9 | Extracellular Space | peptidase |
| matrix metallopeptidase 12 | Mmp12 | -5.709 |  | Extracellular Space | peptidase |
| myeloperoxidase | Mpo | 3.709 |  | Cytoplasm | enzyme |
| murine retrovirus integration site 1 homolog | Mrvi1 | 1.208 | 23 | Cytoplasm | other |
| mucin 6, oligomeric mucus/gel-forming | Muc6 | -3.022 | 10 | Extracellular Space | other |
| MYB proto-oncogene like 1 | Mybl1 | 2.745 | 11 | Nucleus | transcription regulator |
| MYC proto-oncogene, bHLH transcription factor | Myc | 1.578 |  | Nucleus | transcription regulator |
| myosin heavy chain 1 | Myh1 | -1.87 | 14 | Plasma Membrane | enzyme |
| myosin heavy chain 8 | Myh8 | -3.832 | 14 | Cytoplasm | enzyme |
| myosin heavy chain 14 | Myh14 | -2.626 | 14 | Extracellular Space | enzyme |
| myosin VI | Myo6 | 1.276 | 6 | Cytoplasm | other |
| N-6 adenine-specific DNA methyltransferase 1 | N6amt1 | 1.099 |  | Cytoplasm | enzyme |
| napsin A aspartic peptidase | Napsa | -1.418 |  | Extracellular Space | peptidase |
| neuron navigator 2 | Nav2 | -1.525 | 20 | Nucleus | other |
| natural cytotoxicity triggering receptor 1 | Ncr1 | 1.335 |  | Plasma Membrane | transmembrane receptor |
| neural precursor cell expressed, developmentally down-regulated 9 | Nedd9 | 1.235 | 24 | Nucleus | other |
| nidogen 2 | Nid2 | 1.269 | 13 | Extracellular Space | other |
| NK2 homeobox 1 | Nkx2-1 | -1.987 |  | Nucleus | transcription regulator |
| nucleophosmin 1 | Npm1 | 1.338 | 2 | Nucleus | transcription regulator |
| nuclear protein 1, transcriptional regulator | Nupr1 | -2.25 | 17 | Nucleus | transcription regulator |
| ornithine decarboxylase 1 | Odc1 | 1.473 | 17 | Cytoplasm | enzyme |
| prolyl 4-hydroxylase subunit beta | P4hb | -2.446 |  | Cytoplasm | enzyme |
| poly(A) binding protein cytoplasmic 1 | Pabpc1 | 1.976 | 8 | Cytoplasm | translation regulator |
| poly(A) binding protein cytoplasmic 4 | Pabpc4 | 2.079 | 10 | Cytoplasm | translation regulator |
| 3'-phosphoadenosine 5'-phosphosulfate synthase 2 | Papss2 | 1.121 | 13 | Cytoplasm | enzyme |
| poly(ADP-ribose) polymerase 1 | Parp1 | 1.052 |  | Nucleus | enzyme |
| poly(ADP-ribose) polymerase family member 8 | Parp8 | 1.005 | 15 | Other | other |
| proliferating cell nuclear antigen | Pcna | 1.486 | 7 | Nucleus | enzyme |
| programmed cell death 4 | Pdcd4 | 1.586 |  | Nucleus | other |
| phosphodiesterase 2A | Pde2a | 1.466 | 6 | Cytoplasm | enzyme |
| pleckstrin homology like domain family A member 1 | Phlda1 | -1.007 |  | Cytoplasm | other |
| polymeric immunoglobulin receptor | Pigr | -1.026 |  | Plasma Membrane | transporter |
| phospholipase C beta 4 | Plcb4 | 1.164 |  | Cytoplasm | enzyme |
| plectin | Plec | -1.25 | 9 | Cytoplasm | other |
| plastin 1 | Pls1 | 1.281 |  | Plasma Membrane | other |
| plexin B1 | Plxnb1 | -2.91 |  | Plasma Membrane | transmembrane receptor |
| DNA polymerase epsilon, catalytic subunit | Pole | 2.642 | 7 | Nucleus | enzyme |
| cytochrome p450 oxidoreductase | Por | -1.31 |  | Cytoplasm | enzyme |
| PR/SET domain 9 | Prdm9 | 1.039 | 2 | Nucleus | enzyme |
| peroxiredoxin 1 | Prdx1 | -1.15 |  | Cytoplasm | enzyme |
| DNA primase subunit 2 | Prim2 | 1.193 | 13 | Nucleus | enzyme |
| protein kinase C beta | Prkcb | 2.177 | 16 | Cytoplasm | kinase |
| protein kinase, cGMP-dependent, type I | Prkg1 | 1.109 |  | Cytoplasm | kinase |
| proline rich 14 like | Prr14l | 1.06 | 3 | Other | enzyme |
| periaxin | Prx | -1.537 |  | Nucleus | other |
| proteasome subunit beta 4 | Psmb4 | -1.873 |  | Cytoplasm | peptidase |
| protein tyrosine kinase 2 beta | Ptk2b | 1.064 | 18 | Cytoplasm | kinase |
| Rap guanine nucleotide exchange factor 5 | Rapgef5 | 1.045 |  | Nucleus | other |
| RB binding protein 8, endonuclease | Rbbp8 | 1.277 | 1 | Nucleus | enzyme |
| RB transcriptional corepressor like 1 | Rbl1 | 1.237 | 7 | Nucleus | transcription regulator |
| REL proto-oncogene, NF-kB subunit | Rel | 1.517 |  | Nucleus | transcription regulator |
| Rh associated glycoprotein | Rhag | 5.607 |  | Plasma Membrane | peptidase |
| ribonuclease A family member 1, pancreatic | Rnase1 | 10.119 |  | Extracellular Space | enzyme |
| Rho associated coiled-coil containing protein kinase 1 | Rock1 | 1.635 |  | Cytoplasm | kinase |
| ROS proto-oncogene 1, receptor tyrosine kinase | Ros1 | -3.123 |  | Plasma Membrane | kinase |
| repetin | Rptn | -2.759 |  | Cytoplasm | other |
| ribonucleotide reductase catalytic subunit M1 | Rrm1 | 2.041 |  | Nucleus | enzyme |
| ribonucleotide reductase regulatory subunit M2 | Rrm2 | 4.081 |  | Nucleus | enzyme |
| runt related transcription factor 1 | Runx1 | 2.148 |  | Nucleus | transcription regulator |
| RUNX1 translocation partner 1 | Runx1t1 | 1.612 | 18 | Nucleus | transcription regulator |
| secretoglobin family 1A member 1 | Scgb1a1 | -2.738 |  | Extracellular Space | cytokine |
| Scm polycomb group protein like 4 | Scml4 | 1.449 | 13 | Nucleus | other |
| selenium binding protein 1 | Selenbp1 | -2.818 | 1 | Cytoplasm | other |
| serine incorporator 5 | Serinc5 | 1.458 | 2 | Plasma Membrane | transporter |
| surfactant protein C | Sftpc | -3.264 |  | Extracellular Space | other |
| src kinase associated phosphoprotein 1 | Skap1 | 1.86 |  | Cytoplasm | kinase |
| S-phase kinase associated protein 2 | Skp2 | 1.534 | 7 | Nucleus | enzyme |
| solute carrier family 11 member 1 | Slc11a1 | 1.547 |  | Plasma Membrane | transporter |
| solute carrier family 12 member 6 | Slc12a6 | 1.413 | 4 | Plasma Membrane | transporter |
| solute carrier family 7 member 5 | Slc7a5 | 1.499 |  | Plasma Membrane | transporter |
| structural maintenance of chromosomes 3 | Smc3 | 1.06 | 1 | Nucleus | other |
| sorbin and SH3 domain containing 3 | Sorbs3 | -1.029 |  | Cytoplasm | other |
| SRY-box 17 | Sox17 | 1.219 |  | Nucleus | transcription regulator |
| secreted protein acidic and cysteine rich | Sparc | -1.061 |  | Extracellular Space | other |
| spermatogenesis associated 5 | Spata5 | 1.402 |  | Cytoplasm | other |
| secreted phosphoprotein 1 | Spp1 | -1.92 |  | Extracellular Space | cytokine |
| stabilin 2 | Stab2 | 3.399 | 12 | Plasma Membrane | transmembrane receptor |
| signal transducer and activator of transcription 1 | Stat1 | 1.321 |  | Nucleus | transcription regulator |
| stromal interaction molecule 1 | Stim1 | 1.258 | 9 | Plasma Membrane | ion channel |
| spleen associated tyrosine kinase | Syk | 1.205 |  | Cytoplasm | kinase |
| tumor associated calcium signal transducer 2 | Tacstd2 | -3.041 |  | Plasma Membrane | other |
| TAL bHLH transcription factor 1, erythroid differentiation factor | Tal1 | 3.127 |  | Nucleus | transcription regulator |
| transporter 1, ATP binding cassette subfamily B member | Tap1 | 1.302 | 14 | Cytoplasm | transporter |
| T-box 3 | Tbx3 | -1.135 |  | Nucleus | transcription regulator |
| tissue factor pathway inhibitor | Tfpi | 1.695 |  | Extracellular Space | other |
| thymocyte selection associated family member 2 | Themis2 | 1.751 |  | Other | other |
| TIMP metallopeptidase inhibitor 1 | Timp1 | -5.484 | 15 | Extracellular Space | cytokine |
| thymidine kinase 1 | Tk1 | 2.596 | 17 | Cytoplasm | kinase |
| toll like receptor 1 | Tlr1 | 2.294 |  | Plasma Membrane | transmembrane receptor |
| toll like receptor 9 | Tlr9 | 2.769 |  | Plasma Membrane | transmembrane receptor |
| transmembrane BAX inhibitor motif containing 6 | Tmbim6 | -1.266 | 12 | Nucleus | other |
| transmembrane protein 131 | Tmem131 | 1.321 |  | Extracellular Space | other |
| transmembrane and tetratricopeptide repeat containing 3 | Tmtc3 | 1.258 | 2 | Cytoplasm | other |
| DNA topoisomerase II alpha | Top2a | 3.482 | 7 | Nucleus | enzyme |
| tumor protein p53 | Trp53 | 1.576 | 22 | Nucleus | transcription regulator |
| TPX2, microtubule nucleation factor | Tpx2 | 2.96 | 13 | Nucleus | other |
| TRAF3 interacting protein 3 | Traf3ip3 | 2.135 | 23 | Other | other |
| tripartite motif containing 7 | Trim7 | 1.412 | 2 | Cytoplasm | other |
| TSC complex subunit 2 | Tsc2 | -1.092 |  | Cytoplasm | other |
| translocator protein | Tspo | -1.817 |  | Cytoplasm | transmembrane receptor |
| tubulin alpha 1a | Tuba1a | -1.306 | 17 | Cytoplasm | other |
| tubby like protein 4 | Tulp4 | -1.157 | 22 | Cytoplasm | transcription regulator |
| thymidylate synthetase | Tyms | 2.272 | 7 | Nucleus | enzyme |
| ubiquitin conjugating enzyme E2 C | Ube2c | 5.629 | 7,21 | Cytoplasm | enzyme |
| ubinuclein 1 | Ubn1 | -1.207 | 7 | Nucleus | transcription regulator |
| vascular cell adhesion molecule 1 | Vcam1 | 2.814 | 16 | Plasma Membrane | transmembrane receptor |
| von Willebrand factor | Vwf | -1.86 |  | Extracellular Space | other |
| WAP four-disulfide core domain 2 | Wfdc2 | -4.008 |  | Extracellular Space | other |
| zinc finger MYM-type containing 2 | Zmym2 | 1.263 | 18 | Nucleus | kinase |
| zinc finger protein 43 | Zfp458 | 1.436 | 24 | Nucleus | other |
| zinc finger protein 676 | Zfp273 | 1.268 |  | Nucleus | other |

**Supplementary Table 5** 47 molecules related with CD8^+^ T lymphocyte were identified between *Kras^LA2^* and *miR-301a^-/-^;Kras^LA2^* mice.

| Entrez Gene Name | Ensembl/Gene Symbol - mouse | Expr Log Ratio | Networks | Location | Type(s) |
| --- | --- | --- | --- | --- | --- |
| Rac/Cdc42 guanine nucleotide exchange factor 6 | Arhgef6 | 1.272 | 4 | Cytoplasm | other |
| BCL2, apoptosis regulator | Bcl2 | 1.336 |  | Cytoplasm | transporter |
| B-cell CLL/lymphoma 11A | Bcl11a | 2.756 |  | Nucleus | transcription regulator |
| basic helix-loop-helix family member e40 | Bhlhe40 | -2.02 | 24 | Nucleus | transcription regulator |
| caspase 8 | Casp8 | 1.26 | 16 | Nucleus | peptidase |
| C-C motif chemokine receptor 6 | Ccr6 | 1.648 |  | Plasma Membrane | G-protein coupled receptor |
| CD4 molecule | Cd4 | 2.87 | 9 | Plasma Membrane | transmembrane receptor |
| CD5 molecule | Cd5 | 1.457 | 18 | Plasma Membrane | transmembrane receptor |
| CD19 molecule | Cd19 | 3.004 |  | Plasma Membrane | transmembrane receptor |
| CD74 molecule | Cd74 | 1.244 |  | Plasma Membrane | transmembrane receptor |
| CD8a molecule | Cd8a | 2.41 |  | Plasma Membrane | other |
| CD8b molecule | Cd8b1 | 2.453 |  | Plasma Membrane | other |
| class II major histocompatibility complex transactivator | Ciita | 1.121 |  | Nucleus | transcription regulator |
| coronin 1A | Coro1a | 1.685 | 9 | Cytoplasm | other |
| catenin beta 1 | Ctnnb1 | -3.832 | 10 | Nucleus | transcription regulator |
| C-X-C motif chemokine ligand 10 | Cxcl10 | 3.708 |  | Extracellular Space | cytokine |
| dedicator of cytokinesis 8 | Dock8 | 1.081 |  | Cytoplasm | other |
| GIMAP1-GIMAP5 readthrough | Gimap3 | 2.585 | 21 | Cytoplasm | other |
| high-mobility group nucleosome binding domain 5 | Hmgn5 | 1.27 | 10 | Nucleus | other |
| G protein-coupled receptor 18 | Gpr18 | 1.115 |  | Plasma Membrane | G-protein coupled receptor |
| GRB2-related adaptor protein 2 | Grap2 | 1.751 | 18 | Cytoplasm | other |
| human immunodeficiency virus type I enhancer binding protein 2 | Hivep2 | 1.015 |  | Nucleus | transcription regulator |
| major histocompatibility complex, class I, A | H2-Q4 | 1.232 |  | Plasma Membrane | other |
| major histocompatibility complex, class II, DQ beta 1 | H2-Ab1 | 1.15 |  | Plasma Membrane | other |
| interleukin 2 receptor subunit beta | Il2rb | 2.525 |  | Plasma Membrane | transmembrane receptor |
| interleukin 2 receptor subunit gamma | Il2rg | 1.949 |  | Plasma Membrane | transmembrane receptor |
| IL2 inducible T-cell kinase | Itk | 1.925 |  | Cytoplasm | kinase |
| killer cell lectin like receptor C1 | Klrc1 | 2.049 |  | Plasma Membrane | transmembrane receptor |
| leukocyte associated immunoglobulin like receptor 1 | Lair1 | 1.109 |  | Plasma Membrane | transmembrane receptor |
| lymphocyte cytosolic protein 2 | Lcp2 | 1.05 | 18 | Cytoplasm | other |
| metallothionein 1 | Mt1 | -2.795 |  | Cytoplasm | other |
| metallothionein 2 | Mt2 | -2.08 |  | Other | other |
| NLR family CARD domain containing 5 | Nlrc5 | 1.362 |  | Cytoplasm | transcription regulator |
| PBX homeobox 1 | Pbx1 | -1.481 | 15 | Nucleus | transcription regulator |
| phosphatidylinositol-4,5-bisphosphate 3-kinase catalytic subunit delta | Pik3cd | 1.478 |  | Cytoplasm | kinase |
| protein kinase C theta | Prkcq | 1.332 | 19 | Cytoplasm | kinase |
| protein tyrosine phosphatase, non-receptor type 6 | Ptpn6 | 1.458 |  | Cytoplasm | phosphatase |
| protein tyrosine phosphatase, receptor type C | Ptprc | 1.716 |  | Plasma Membrane | phosphatase |
| RAS guanyl releasing protein 1 | Rasgrp1 | 2.163 |  | Cytoplasm | other |
| ras homolog family member H | Rhoh | 2.092 |  | Plasma Membrane | enzyme |
| transporter 1, ATP binding cassette subfamily B member | Tap1 | 1.302 | 14 | Cytoplasm | transporter |
| transcription factor 7, T cell specific | Tcf7 | 1.451 |  | Nucleus | transcription regulator |
| thymocyte expressed, positive selection associated 1 | Tespa1 | 2.369 | 18 | Cytoplasm | other |
| thymocyte selection associated | Themis | 2.013 |  | Cytoplasm | other |
| thymocyte selection associated high mobility group box | Tox | 1.576 |  | Nucleus | other |
| tumor protein p53 | Trp53 | 1.576 | 22 | Nucleus | transcription regulator |
| vav guanine nucleotide exchange factor 1 | Vav1 | 1.013 | 9 | Nucleus | transcription regulator |
| Rac/Cdc42 guanine nucleotide exchange factor 6 | Arhgef6 | 1.272 | 4 | Cytoplasm | other |

**Supplementary Table 6** The primer used for real-time PCR assay.

|  | Gene Name | Forward sequence (5'-3') | Reverse sequence(5'-3') |
| --- | --- | --- | --- |
| 1 | mIL-6 | cctctggtcttctggagtacc | actccttctgtgactccagc |
| 2 | mTNFα | atgagcacagaaagcatga | agtagacagaagagcgtggt |
| 3 | mIL-17 | tcccctctgtgatctgggaag | agcatcttctcgaccctgaa |
| 4 | mIL-1α | ctctagagcaccatgctacagac | tggaatccaggggaaacactg |
| 5 | mIl-1β | ctccatgagctttgtacaagg | tgctgatgtaccagttgggg |
| 6 | mIFNG | atgaacgctacacactgcatc | ccatccttttgccagttcctc |
| 7 | mIL-10 | ccctttgctatggtgtcctt | tggtttctcttcccaagacc |
| 8 | mIL-22 | tttcctgaccaaactcagca | ctggatgttctggtcgtcac |
| 9 | mIL-23 | cacctccctactaggactcagc | tgggcatctgttgggtct |
| 10 | mGM-CSF | aacctcctggatgacatg | aaattgccccgtagaccc |
| 11 | mIL-4 | aacgaggtcacaggagaagg | tctgcagctccatgagaaca |
| 12 | mTGFβ | gctaccatgccaacttctgt | cgtagtagacgatgggcagt |
| 13 | mVEGF | catcttcaagccgtcctgtgt | ctccagggcttcatcgttaca |
| 14 | mCXCL9 | cttttcctcttgggcatcat | gcatcgtgcattccttatca |
| 15 | mCXCL10 | gctgccgtcattttctgc | tctcactggcccgtcatc |
| 16 | mCCL17 | tgcttctggggacttttctg | gaatggcccctttgaagtaa |
| 17 | mCCL22 | tcttgctgtggcaattcaga | gagggtgacggatgtagtcc |
| 18 | mMMP9 | acgacatagacggcatcca | gctgtggttcagttgtggtg |
| 19 | hItga4 | tcggagccagcatactacc | ccacagcacagacagaagc |
|  | mItga4 | cccaggctacatcgtttttgt | catgaatgggggtaaggatg |
| 20 | hSlc25α21 | gtttgttaggaggtgtaagtgttgt | ccaataatttatcaaactaaacaat |
|  | mSlc25α21 | cgaggtggtaaaagttggcttgc | gctgtcaatcctttgtcgaggc |
| 21 | hAdam23 | ccactcgattccaagggtaa | accagcgatggagcctatta |
|  | mAdam23 | ttccaatggcggtggcacaagt | cctgtttcttccatgatgcagcc |
| 22 | hRunx3 | gcaggcaatgacgagaacta | cagtgatggtcagggtgaaa |
|  | mRunx3 | tggctagacattcctgtggga | cttggttggctgctgttttgt |
| 23 | hBach2 | ctcgagaataccagcttgcatgtaccaa | gcggccgcttatcttcccggaatgtgcttg |
|  | mBach2 | gtcgaaagaggaagctggactg | gaggcaggaaaagttgtccagg |
| 24 | hFam107b | caaggactcgacctgagaag | ctatccacagttccacaactg |
|  | mFam107b | ccgaaatcaccaagacctccac | ctgctttatcacttggtctcgtc |
| 25 | hPten | tgagttccctcagccgttacct | gaggtttcctctggtcctggta |
| 26 | hβ-actin | agcacggcatcgtcaccaactg | gagctggaagcagccgtggcc |
| 27 | mβ-actin | ggctatgctctccctcacg | cttctctttgatgtcacgcacg |
