# Supplementary material for: miR-301a promotes lung tumorigenesis by suppressing Runx3
Source: Mol Cancer. 2019 May 23;18:99. doi: 10.1186/s12943-019-1024-0 (PMC6532219; doi:10.1186/s12943-019-1024-0)
Supplement: Supplementary file 2 — Figure S1. IPA Interaction networks analysis of significant DEGs in lung tissue between KrasLA2 and miR-301a−/−KrasLA2 mice and identified IFNG and CTNNB1 as the central node in the top regulated network. Figure S2. Runx3 is a direct target of miR-301a. Figure S3. The mRNA and protein expression of 5 genes in NSCLC cell lines. Figure S4. The effects of PTEN on cell proliferation in A549 and lung tumorigenesis. Figure S5. The effects of miR-301a on cell proliferation and apoptosis in mouse xenografts (DOCX 4049 kb) [file 12943_2019_1024_MOESM2_ESM.docx]

**Supplementary Figures for**

***Xun Li* et al, “miR-301a Promotes Lung Tumorigenesis by Suppressing Runx3”**

Table of Contents

**Supplementary Figure 1. IPA Interaction networks analysis of significant DEGs in lung tissue between *Kras^LA2^* and *miR-301a^-/-^Kras^LA2^* mice and identified IFNG and CTNNB1 as the central node in the top regulated network ……………………... 2**

**Supplementary Figure 2. Runx3 is a direct target of miR-301a ……………… 3**

**Supplementary Figure 3. The mRNA and protein expression of 5 genes in NSCLC cell lines …………………….... ………………………………………………… 4**

**Supplementary Figure 4. The effects of PTEN on cell proliferation in A549 and lung tumorigenesis ……………………………………………………………………....... 5**

**Supplementary Figure 5. The effects of miR-301a on cell proliferation and apoptosis in mouse xenografts ……………………………………………………………....... 6**

**
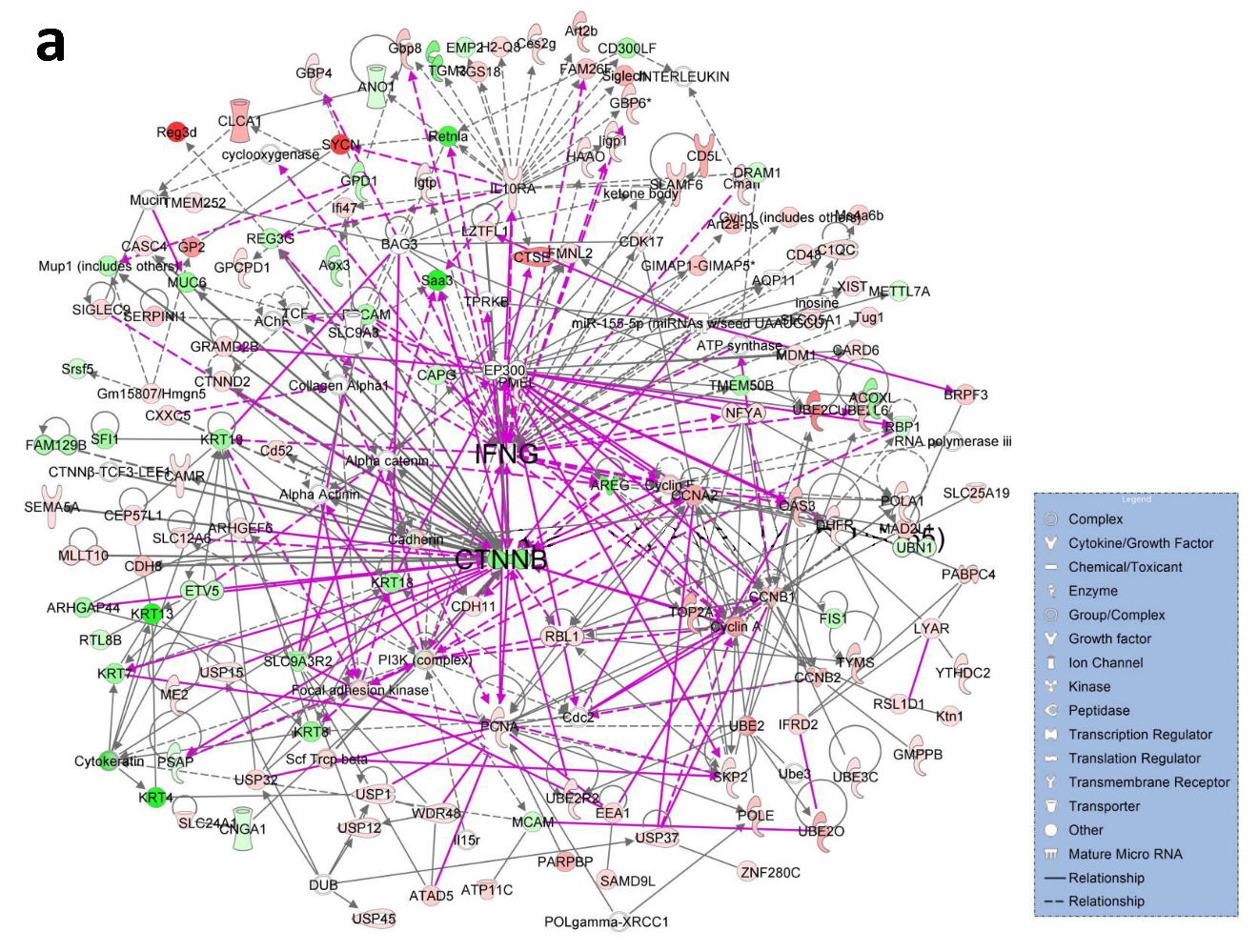
**

**Supplementary Figure 1. IPA Interaction networks analysis of significant DEGs in lung tissue between *Kras^LA2^* and *miR-301a^-/-^Kras^LA2^* mice and identified IFNG and CTNNB1 as the central node in the top regulated network.**  (a) Top five networks were analyzed by IPA and merged into one figure. Downregulated genes (green) and upregulated genes (red) were identified in the network. Genes in the network without any color were analyzed by IPA to show the potential connectivity.


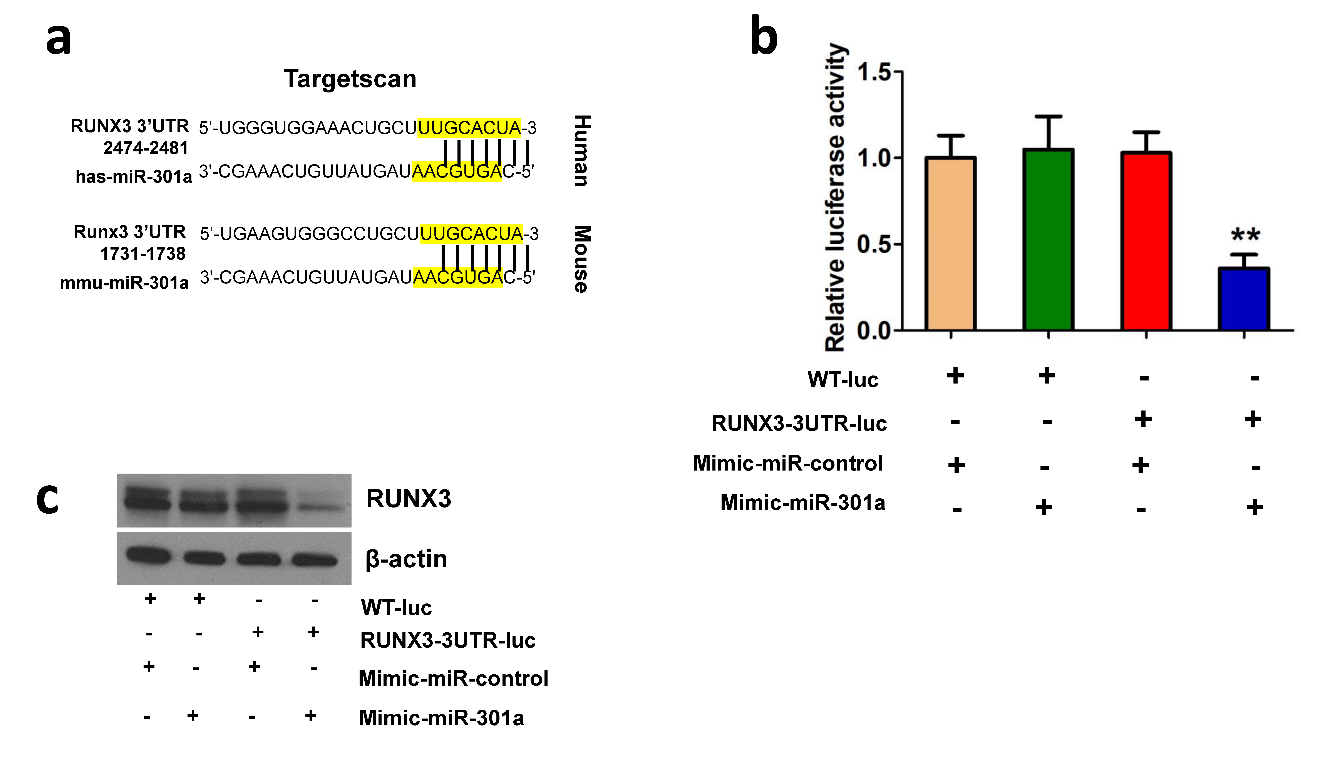


**Supplementary Figure 2.** **Runx3 is a direct target of miR-301a.** (a) Prediction of major interference sites between miR-301a and RUNX3 mRNA 3’UTR by using Targetscan. (b) Luciferase activity in 293T cells transfected with the indicated luciferase reporter with either an miR-301a mimic or control oligonucleotide. (c) Western blot analysis of RUNX3 expression in 293T cells transfected with the indicated luciferase reporter with either an miR-301a mimic or control oligonucleotide. Values represented the mean ± s.d. of three independent experiments. ***P* < 0.01 indicates a significant difference between the indicated groups (one-way analysis of variance (ANOVA) in b).

**
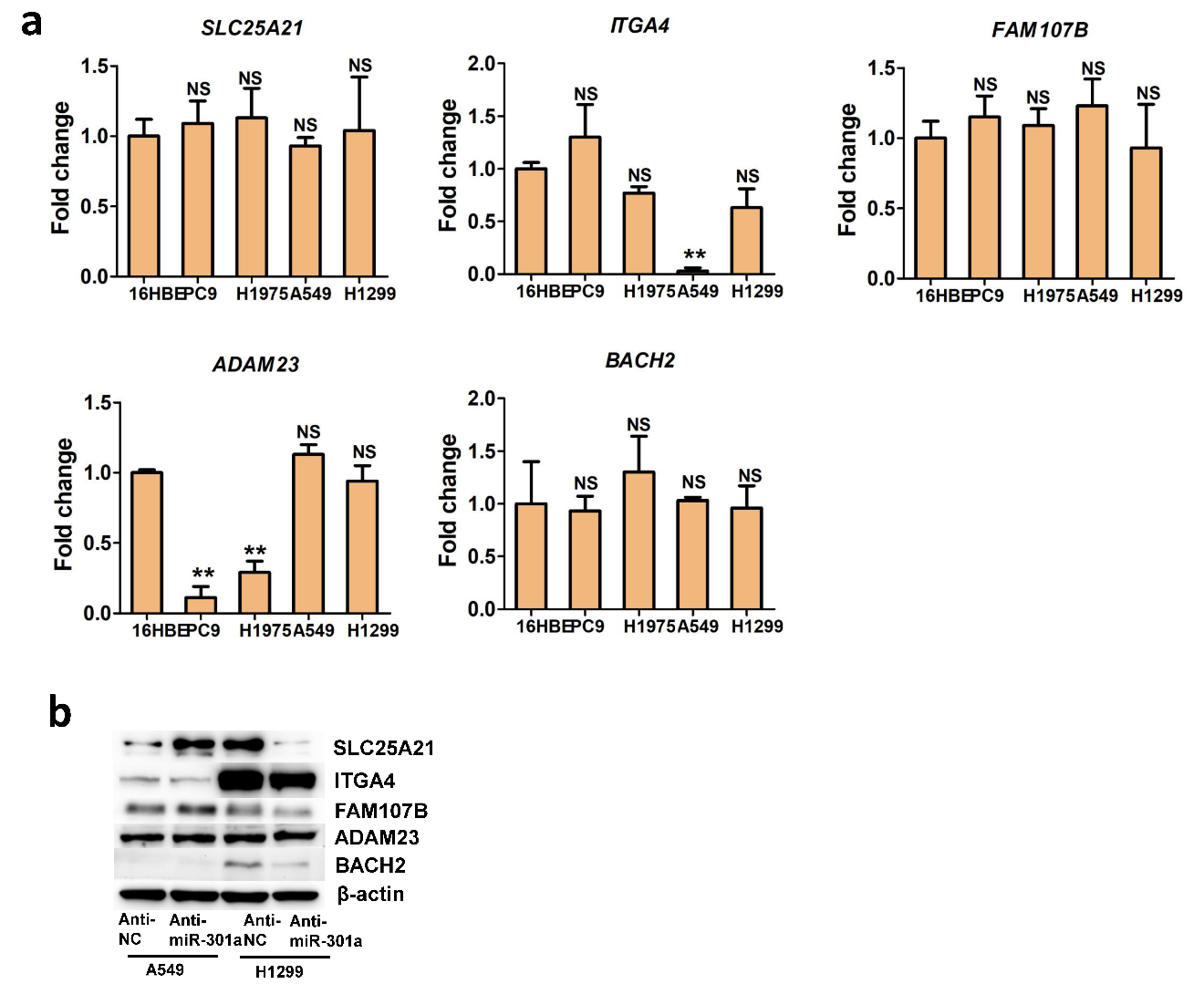
**

**Supplementary Figure 3. The mRNA and protein expression of 5 genes in NSCLC cell lines.** (a) qPCR analyses of SLC25A21, ITGA4, FAM107B, ADAM23 and BACH2 expression between normal bronchial cell line (16HBE) and NSCLC cell lines (PC9, H1975, A549 and H1299). Total RNAs were extracted from those five cell lines and performed by qPCR analysis. (b) Western blotting analyses of SLC25A21, ITGA4, FAM107B, ADAM23 and BACH2 expression in A549 and H1299 cells transfected with anti-negative control (Anti-NC) and LNA-anti-miR-301a (Anti-miR-301a). Values represented the mean ± s.d. of three independent experiments. ***P* < 0.01 indicates a significant difference between the noncancerous bronchial cell line and NSCLC cell lines (one-way analysis of variance (ANOVA) in a). NS, not significant.

**
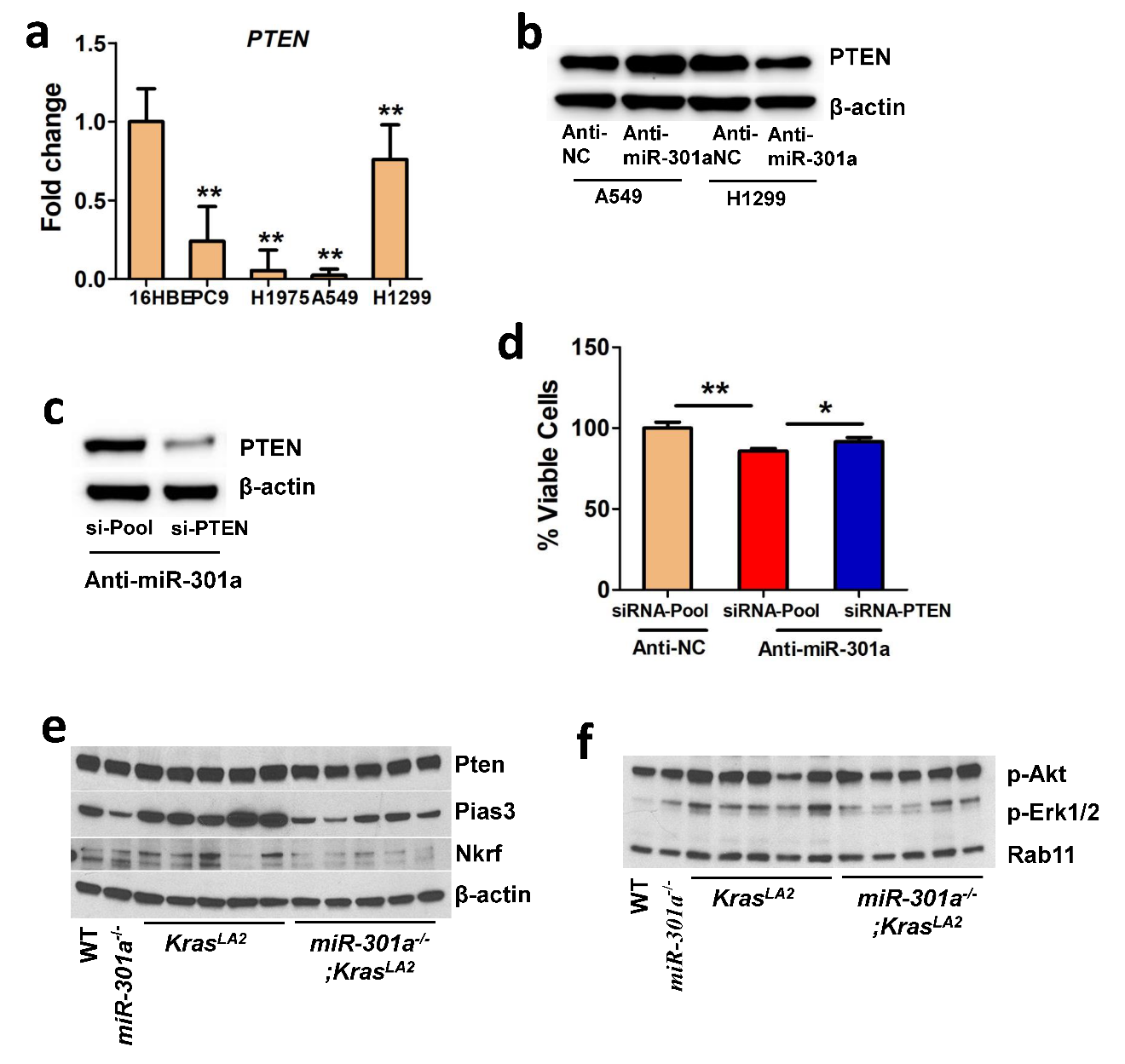
**

**Supplementary Figure 4. The effects of PTEN on cell proliferation in A549 and lung tumorigenesis.** (a) qPCR analyses of *PTEN* expression between normal bronchial cell line (16HBE) and NSCLC cell lines (PC9, H1975, A549 and H1299). Total RNAs were extracted from those five cell lines and performed by qPCR analysis. (b) Western blotting analyses of PTEN expression in A549 and H1299 cells transfected with anti-negative control (Anti-NC) and LNA-anti-miR-301a (Anti-miR-301a). (c) Western blotting shows the efficacy of siRNA knockdown in A549 co-transfected with LNA-anti-miR-301a and SiRNA-PTEN or SiRNA-control. (d) A549 cells were transfected with anti-NC and siRNA control (SiRNA-Pool), Anti-miR-301a and SiRNA-Pool, and Anti-miR-301a and SiRNA-PTEN. After 48h of transfection, cell viability of A549 cells were determined by cell counting kit-8. (e) The expression level of Pten, Pias3 and Nkrf from lung tumor tissues of *Kras^LA2^* (n=5) and *miR-301a^-/-^Kras^LA2^* (n=5) mice at 9 weeks of age were determined by western blotting. (f) The expression level of phospho-Akt, phospho-Erk1/2 and Rab11(Control) from lung tumor tissues of *Kras^LA2^* (n=5) and *miR-301a^-/-^Kras^LA2^* (n=5) mice at 9 weeks of age were determined by western blotting. ***P* < 0.01 or **P* < 0.05 indicates a significant difference between the indicated groups (one-way analysis of variance (ANOVA) in a and d). NS, not significant.


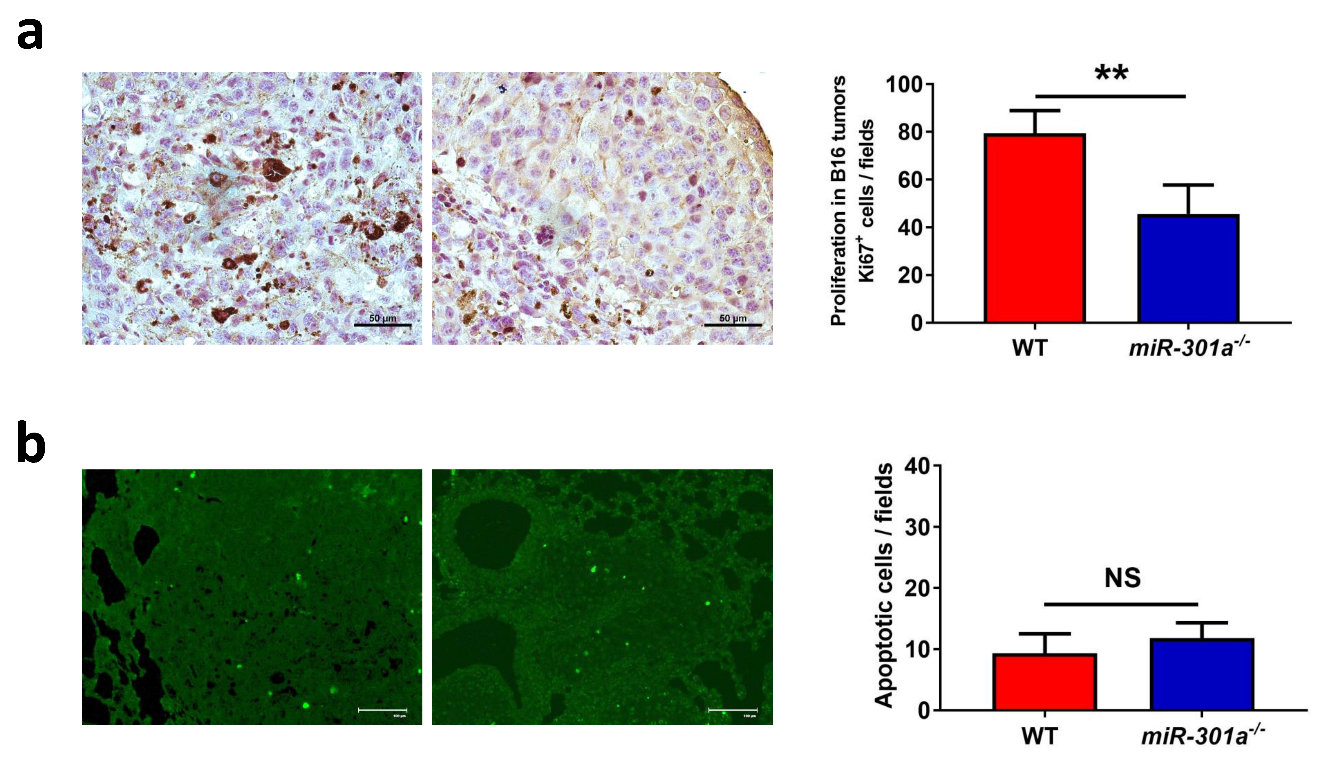


**Supplementary Figure 5. The effects of miR-301a on cell proliferation and apoptosis in mouse xenografts.** (a) Cell proliferation from lung tumors of WT and *miR-301a^-/-^* mice implanted with B16 tumor cells. (b) Quantification of apoptotic cells from lung tumors of WT and *miR-301a^-/-^* mice implanted with B16 tumor cells.
